# FlavonoidSearch: A system for comprehensive flavonoid annotation by mass spectrometry

**DOI:** 10.1038/s41598-017-01390-3

**Published:** 2017-04-28

**Authors:** Nayumi Akimoto, Takeshi Ara, Daisuke Nakajima, Kunihiro Suda, Chiaki Ikeda, Shingo Takahashi, Reiko Muneto, Manabu Yamada, Hideyuki Suzuki, Daisuke Shibata, Nozomu Sakurai

**Affiliations:** 1 Kazusa DNA Research Institute, Kazusa-kamatari 2-6-7, Kisarazu, Chiba 292-0818 Japan; 2 Kyoto University, Graduate School of Agriculture, Gokasho, Uji, Kyoto 611-0011 Japan; 3 KAGOME CO., LTD., Nishitomiyama 17, Nasushiobara, Tochigi 329-2762 Japan

## Abstract

Currently, in mass spectrometry-based metabolomics, limited reference mass spectra are available for flavonoid identification. In the present study, a database of probable mass fragments for 6,867 known flavonoids (FsDatabase) was manually constructed based on new structure- and fragmentation-related rules using new heuristics to overcome flavonoid complexity. We developed the FlavonoidSearch system for flavonoid annotation, which consists of the FsDatabase and a computational tool (FsTool) to automatically search the FsDatabase using the mass spectra of metabolite peaks as queries. This system showed the highest identification accuracy for the flavonoid aglycone when compared to existing tools and revealed accurate discrimination between the flavonoid aglycone and other compounds. Sixteen new flavonoids were found from parsley, and the diversity of the flavonoid aglycone among different fruits and vegetables was investigated.

## Introduction

Flavonoids are secondary metabolites derived from plants, and these compounds are a major target for mass spectrometry (MS)-based metabolomics because of their potential health benefits and functions in plants^1^. To date, >7,000 flavonoids have been identified, with many flavonoids showing antioxidant, anti-inflammatory, anti-cancer, estrogen-like and signal transduction activities^2^. However, because of the limited availability of authentic standards for peak identification in tandem mass spectrometry (MS/MS or MS^n^), comprehensive identification or annotation of flavonoids remains a challenge^3, 4^. Modification of the structure of a flavonoid can occur in the soil, during food fermentation processes and in the body (e.g. in the intestine or liver)^5^. This results in a high diversity of flavonoids, and a comprehensive and high-throughput technique is required for their annotation.

Virtual mass spectra can be used to overcome mass spectral library limitations^6^. A spectral library constructed based on compound group-specific rules of fragmentation in collision-induced dissociation (CID) has been constructed for the lipidomes^7^; however, to date, this strategy has not been applied to other compound groups. In the case of flavonoids, this is because their structural features result in highly complex fragmentation^8–11^. Flavonoids have a core structure called the aglycone, which contains a diphenylpropane backbone (C_6_-C_3_-C_6_) and various types and numbers of substituents (e.g. glycosyl and acyl groups)^12^. This is similar to lipids, which have head groups containing various acyl residues^7^. A major difference between flavonoids and lipids is that fragmentation of the flavonoid aglycone is largely influenced by the substituents, whereas fragmentation of lipids is not. For flavonoids containing substituents with low degrees of dissociation, such as *C*-prenyl groups, the fragment formed from the backbone includes the substituents^13^. By contrast, for flavonoids with substituents that have high degrees of dissociation, such as *O*-glycosides, fragmentation of these substituents occurs during the first stage of MS/MS (MS^2^)^9^. In the subsequent stage (MS^3^), fragments are formed with the remaining aglycone and any substituents with low degrees of dissociation. Therefore, to predict the mass fragments for flavonoid fragmentation, the structural unit that fragments with the aglycone moiety needs to be considered. In the present study, we refer to these units as ‘MSMS-aglycones’. Because there are many more structural variants (3,678) of the MSMS-aglycones than the lipid head groups (~30)^7^, an enormous number of manual calculations and curations would be required to predict the virtual mass fragments for each of the MSMS-aglycones. However, the completed dataset would allow comprehensive annotation of flavonoids, and the resulting lists of MSMS-aglycones and substituents would be an important resource for discovering novel flavonoids.

In the present study, we developed a system for annotating flavonoids (FlavonoidSearch), which consists of a database of virtual mass fragments (FsDatabase) predicted from 6,867 known flavonoid structures in one of the largest flavonoid databases (metabolomics.jp) and a computational tool (FsTool) to search the database.

## Results

### Construction of a database of virtual mass fragments (FsDatabase)

**Figure 1:**
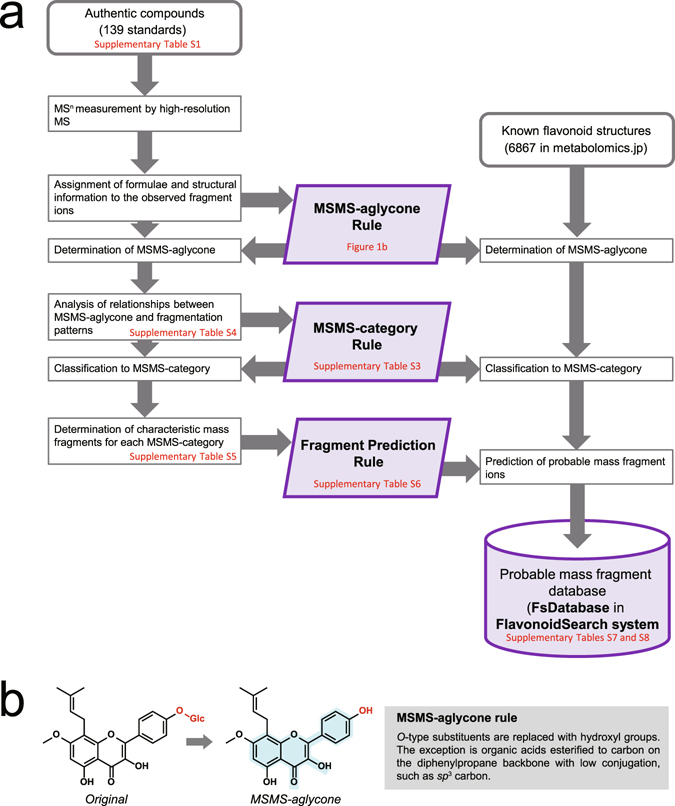
Construction of the probable mass fragment database of flavonoids (FsDatabase) for use in the FlavonoidSearch system. (**a**) Schematic outline of the construction of the FsDatabase. We analysed 139 authentic flavonoids and used the data to develop the following three rules: (1) the MSMS-aglycone rule to define the base structural unit of fragmentation (MSMS-aglycone); (2) the MSMS-category rule to define fragmentation patterns that were characteristic of flavonoid classes (Fig. 1b); and (3) the fragment prediction rule to identify probable fragments. These rules were used to create virtual fragments from 6,867 known flavonoids based on their chemical structures. References to related figures and tables are given in red. (**b**) The MSMS-aglycone rule (in the grey box) and an example of an MSMS-aglycone (right). According to the rule, the *O*-glucosyl group in the original structure (left) was replaced with a hydroxyl group. The C_6_-C_3_-C_6_ backbone structure is highlighted in pale blue.

**Figure 2:**
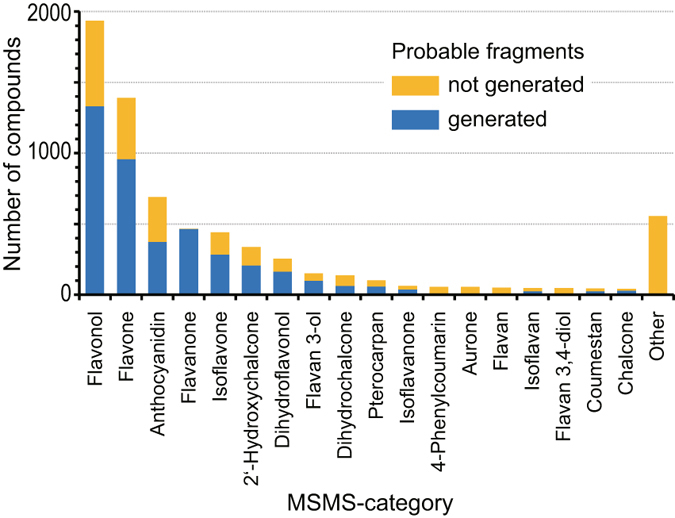
Coverage of flavonoids predictable by FlavonoidSearch. For the 6,867 known flavonoids, those with probable mass fragments generated in FsDatabase are shown in blue.

A schematic description of the construction of the FsDatabase is shown in Fig. 1. To confirm the existing knowledge of flavonoid fragmentation^8–11^ and to investigate new fragmentation information, we analysed 139 commercially available flavonoid standards using high-resolution Fourier transform (FT) ion cyclotron resonance MS. Chemical formulae, with or without structural information, were assigned to 1,080 fragment ions using their accurate masses and existing knowledge of fragmentation (Supplementary Table S2 and MS-MS Fragment Viewer: http://webs2.kazusa.or.jp/msmsfragmentviewer/). Using the assignment results, we constructed a ‘MSMS-aglycone rule’ to determine MSMS-aglycone moieties in the original 6,867 structures (Fig. 1b). Using this rule, all *O*-glycosides are replaced with hydroxyl groups, and hydroxyl, methoxyl and *C*-type substituents bonded to the backbone are retained in the MSMS-aglycones. Next, we investigated the relationships between fragmentation and structural features of the MSMS-aglycones with reference to existing knowledge. Flavonoids with common substructures are known to show similar fragmentation. However, we identified that some flavonoids that would traditionally be placed in the same class showed different fragmentation to other flavonoids in that class. Therefore, we developed a new classification system (‘MSMS-category’) and a rule to determine the MSMS-category from the MSMS-aglycone structure using the C_6_-C_3_-C_6_ backbone structure and type and position of substituents that could affect fragmentation (Supplementary Table S3). In addition, we found that the fragmentation patterns for fragments derived from C-ring cleavage, small neutral loss and neutral loss of substituents on A- and/or B-rings were related to the index of hydrogen deficiency (IHD) of the backbone of the MSMS-aglycone (Supplementary Table S4). The general trends observed for these fragment ions are referred to as ‘IHD heuristics’ (see Supplementary Methods for details). Based on these findings, we selected characteristic fragments for MSMS-categories where there were more than two standards for analysis (Supplementary Table S5) and constructed a rule to predict possible fragment ions for each MSMS-category using the IHD heuristics (Supplementary Table S6). Finally, we applied the three rules to the 6,867 flavonoid structures registered in metabolomics.jp and constructed the FsDatabase of predicted fragments. FsDatabase contains fragments for 4,119 out of the 6,867 flavonoid species, including 1,489 out of 3,678 unique MSMS-aglycones classified into 14 out of 124 MSMS-categories (Supplementary Table S7). The *O*-substituents in the known flavonoids are listed in Supplementary Table S8. The FsDatabase covers the major flavonoid aglycones (Fig. 2).

### Evaluation of the FsDatabase for flavonoid identification

**Figure 3:**
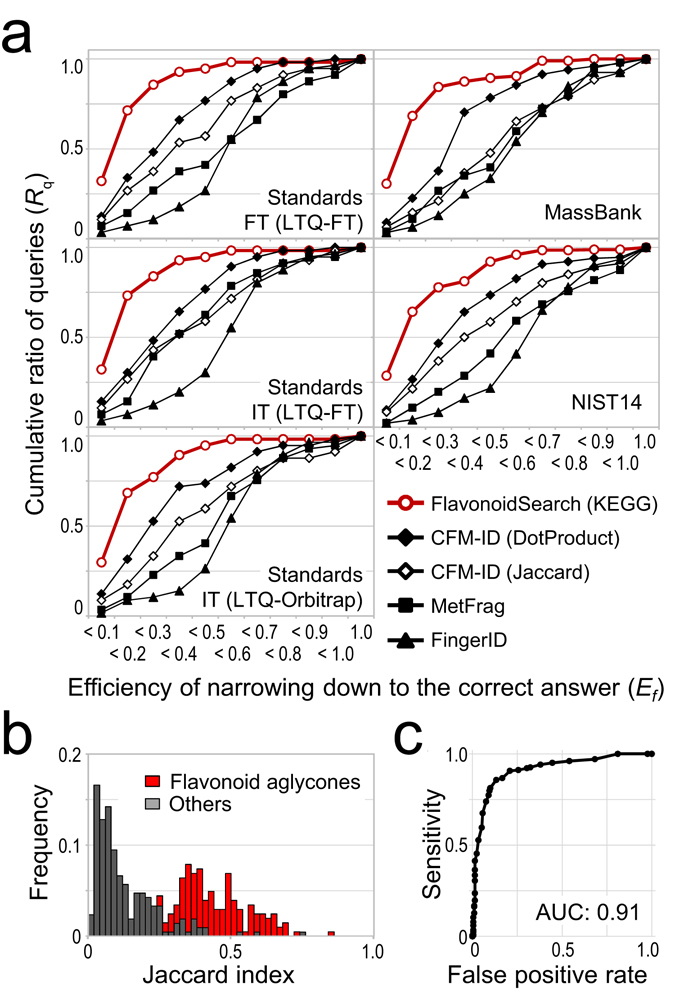
Accuracies and discrimination power of the FlavonoidSearch system. (**a**) The accuracy of each search tool was evaluated using the area under the cumulative curve (AUCc) for a plot of cumulative ratio of queries (*Y*-axis) to the efficiency of narrowing down to the correct answer (*X*-axis). The cumulative curve will move closer to the upper left-hand corner of the figure when highly narrowed-down results are obtained for a high number of queries. Therefore, a high AUCc is indicative of high accuracy. Results are shown for data obtained in-house for flavonoid aglycones using ion trap (IT) and Fourier transform (FT) mass spectrometry (MS) of LTQ-FT and IT of LTQ-Orbitrap and spectra retrieved from MassBank and NIST14 searched with the Kyoto Encyclopedia of Genes and Genomes (KEGG) database. (**b**) Differences in the frequency distributions of Jaccard indices for flavonoid aglycones (red bars) and other compounds (grey bars). The frequency represents the ratio of records with a range of Jaccard indices to all records that had a Jaccard index >0. (**c**) A receiver operating characteristic curve of the discrimination test by a binary classification in (**b**), yielding an area under the curve (AUC) of 0.91. Results obtained with ITFT data from NIST14 are shown in (**b**) and (**c**).

Accuracy of flavonoid identification using the FsDatabase was evaluated using a Java-based tool (FsTool) to query the database (available at http://www.kazusa.or.jp/komics/software/FlavonoidSearch/). The accuracy obtained with this tool was compared with that obtained from existing tools, including CFM-ID^14^, FingerID^15^ and MetFrag^16^. FsTool efficiently narrowed down the correct answer to within the top 20% among a set of isobaric candidates for 70% of queries conducted with both low- (ion trap, IT) and high- (FT) resolution data used for FsDatabase construction (Fig. 3a, Supplementary Fig. S2 and Supplementary Tables S9–S12). Among the search tools, FsTool showed the highest accuracy for data obtained from many mass platforms (>50 spectrometer types), including in-house data from a linear IT combined with an Orbitrap (LTQ-Orbitrap, Thermo Fisher Scientific) and data from MassBank^17^ and NIST14 Mass Spectral Library (Ver. June 2014, National Institute of Standards and Technology, Gaithersburg, MD) (Supplementary Table S16). Surprisingly, good results were also observed for data from higher-energy collisional dissociation (HCD), which was not used for the construction of the FsDatabase in the present study.

### Evaluation of the discrimination power

As shown in Fig. 3b, flavonoid aglycones showed higher Jaccard indices than other compounds. We evaluated the power of FsTool for discriminating between flavonoid aglycones and other compounds using MassBank and NIST data. The result was classified as positive (flavonoid aglycone) with the Jaccard index as the hit score was >0. A high specificity (>0.95) was observed (Supplementary Table S13). Moderate sensitivity over a wide range (0.77–0.93) and differences in the Jaccard index at maximum accuracy (Supplementary Table S15, Supplementary Fig. S3) implied that the discrimination ability was platform dependent. Nevertheless, the range for the area under the curve (AUC) for the receiver operator characteristic curve was 0.82–0.98 (Fig. 3c, Supplementary Table S15, Supplementary Fig. S3). HCD data from NIST comprised the single outlier, which had an AUC of 0.67. These results suggest that the Jaccard index is useful for determining if a compound is a flavonoid aglycone. One major false positive was noted, which involved misidentification of *O*-glycosides (not MSMS-aglycones) as their isobaric *C*-glycosides because of traces of fragments from neutral loss. This issue should be investigated in future research.

### Application of the FlavonoidSearch system to practical metabolome data

**Figure 4:**
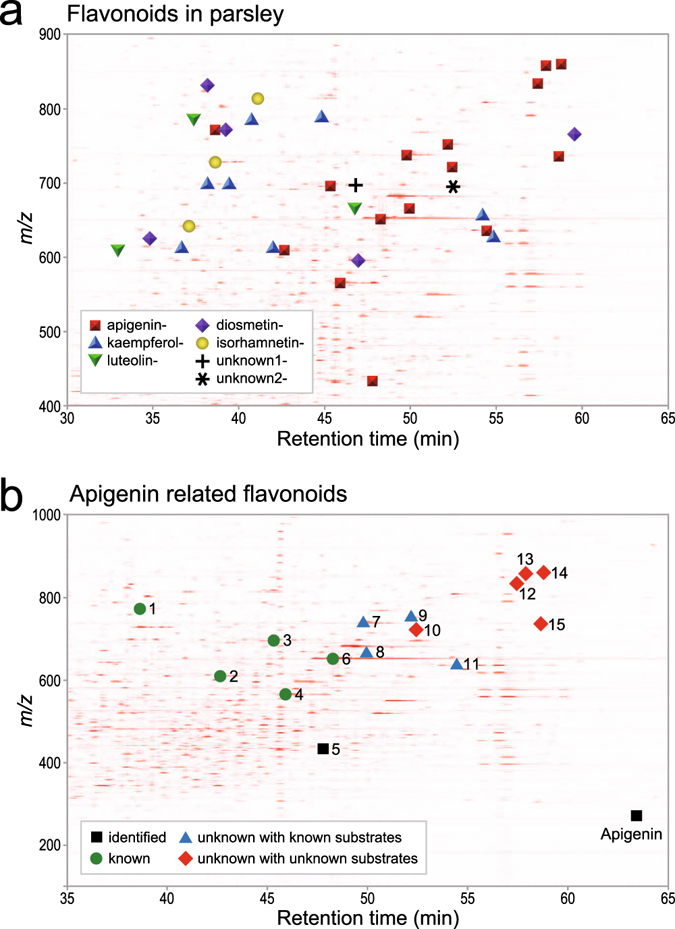
Flavonoids in parsley samples. (**a**) Flavonoids in parsley annotated by FlavonoidSearch (Jaccard index >0.3) and manual curation. Peaks of derivatives of characteristic aglycones in parsley (apigenin derivatives, red squares; kaempferol derivatives, blue triangles; luteolin derivatives, green inverted triangles; diosmetin derivatives, purple diamonds and isorhamnetin derivatives, yellow circles) and two unknown aglycones (C_16_H_13_O_7_^+^, plus symbol; and C_17_H_15_O_6_^+^, asterisk) are represented on a two-dimensional (2D) mass chromatogram. The peak positions on an overall view of the 2D mass chromatogram is shown in Supplementary Fig. S4. (b) Apigenin-related compounds annotated in parsley. Peaks identified using authentic compounds (black squares), known derivatives (green circles), unknown derivatives with combinations of known *O*-substituents (blue triangles) and unknown derivatives with unknown substituents (red diamonds) are represented on a 2D mass chromatogram. The numbers correspond to the peak numbers shown in Supplementary Table S17.

**Figure 5:**
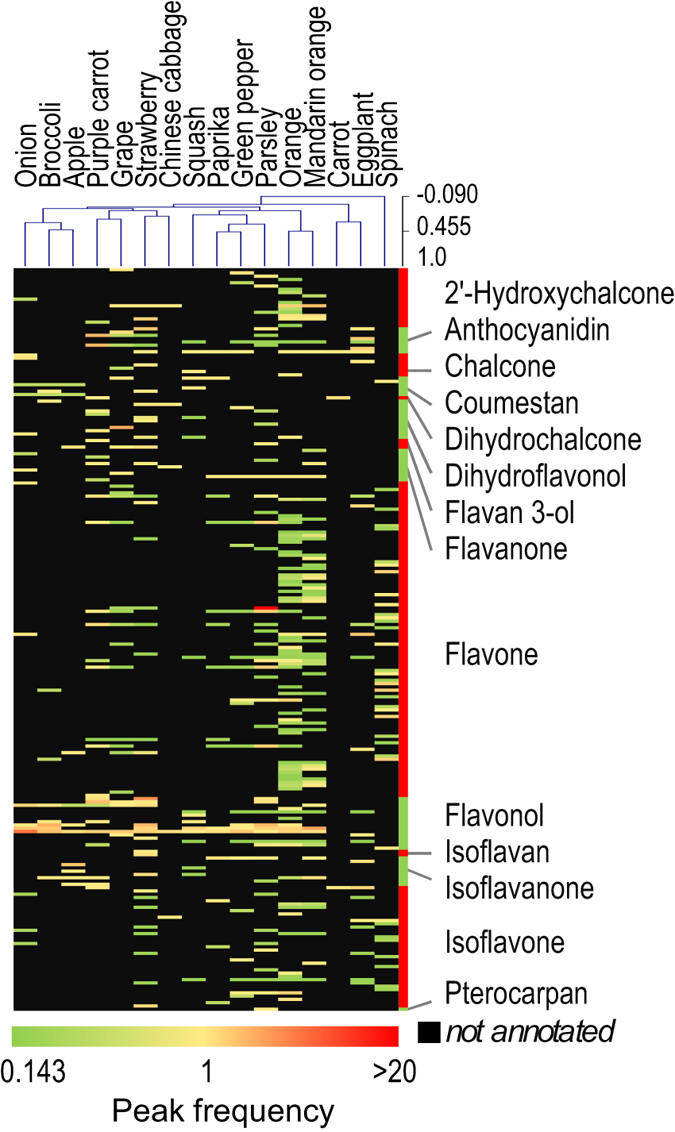
The diversity of flavonoid aglycones in 16 plant samples. A hierarchical cluster analysis for annotated flavonoid aglycones in 16 plant samples was performed. MS^3^ spectra were obtained semi-comprehensively by a data-dependent acquisition from the samples. Numbers of peaks annotated to the symbolised name of the MSMS-aglycones by FlavonoidSearch (Jaccard index >0.3) are represented as ‘peak frequency’ (see Methods).

To investigate the application of FlavonoidSearch to practical metabolome data, we examined semi-comprehensive MS^3^ data from parsley. Many flavonoid candidates were detected, including derivatives of major known aglycones (apigenin, luteolin, kaempferol, diosmetin and isorhamnetin) in parsley (Fig. 4a, Supplementary Fig. S4)^18, 19^. We confirmed that the annotations for three compounds, namely apigenin, apigenin-7-*O*-glucoside and apigenin-6,8-di-*C*-glucoside, which were not used for construction of FsDatabase, were correct by identification using available authentic compounds. We found that many parsley peaks showed hits on the FsDatabase using their MS^n^ spectra, but not on other natural product databases using mass values of the peaks. Using the *O*-substituent list (Supplementary Table S8), eight of the compounds were annotated as novel flavonoids with new combinations of known MSMS-aglycones and known *O*-substituents (Fig. 4b, Supplementary Table S17). Furthermore, we discovered five unknown substituents attached to known aglycones. Interestingly, an *O*-substituent (C_10_H_16_O_5_ as neutral loss) was attached to both apigenin and diosmetin (Supplementary Table S18). An additional *O*-substituent (C_17_H_22_O_7_ as neutral loss) attached to apigenin has been annotated in a kaempferol-derivative in tomato^20^. A search in SciFinder did not yield any possible structures, suggesting that these compounds are novel flavonoids. These compounds were present at low levels; therefore, we could not perform experiments to determine their structures in this study. Careful analysis of the fragmentation patterns also revealed unknown MSMS-aglycones in parsley. One aglycone (C_16_H_13_O_7_^+^) was isomeric to isorhamnetin, whereas another (C_17_H_15_O_6_^+^) was speculated to be a methoxylated form of kaempferol, luteolin or diosmetin. We investigated the diversity of aglycones in 16 types of vegetables and fruits (Fig. 5 and Supplementary Table S19). Although there were differences among the sampling tissues, quercetin- and isorhamnetin-related flavonols were common in most of the samples, and some trends between the types of substituents for anthocyanidins and flavones were observed. The findings on quercetin derivatives are consistent with their wide distribution in the plant kingdom^21, 22^.

## Discussion

FlavonoidSearch enables the comprehensive and high-throughput annotation of flavonoids without the need for expert knowledge of fragmentation or the structures of flavonoids. This system will contribute to the further understanding and application of flavonoids. We found 16 novel flavonoids in parsley using this system in combination with the *O*-substituent list (Supplementary Table S8), which suggests that many minor flavonoids have been overlooked in earlier studies using conventional ultraviolet detectors. Remarkably, parsley and tomato showed the same unknown *O*-substituents, implying that these plants share previously unknown pathways of transformation of flavonoids (e.g. biosynthesis, biodegradation and/or abiotic transformation). Our system could be used to uncover the dynamics of the ‘flavonome’ and will also contribute to discovery and use of the biosynthetic gene resources for the bioengineering of functional flavonoids.

One limitation of the system is the requirement for comprehensive MS^3^ spectra because MS^3^ spectra cannot be obtained by all mass spectrometry techniques. One solution is the use of pseudo-MS^3^ spectra, which is obtained from a combination of in-source fragmentation with high cone voltage and subsequent CID or HCD fragmentation in conventional mass spectrometer^23^. The aglycone moieties of flavonoids were identified by comparing pseudo-MS^3^ spectra with the MS^2^ spectra of the authentic standards of the aglycones^3, 24–26^. Therefore, it will become possible to apply FlavonoidSearch to pseudo-MS^3^ spectra. The evaluation of the accuracy of flavonoid identification is necessary to determine whether FlavonoidSearch shows good performance in the identification of fragment ions of the aglycone moiety in a pseudo-MS^3^ spectrum. We expect that FlavonoidSearch would also be applicable to a pseudo-MS^3^ spectrum, because FlavonoidSearch showed effective identification for various spectra obtained from many mass spectrometers (Supplementary Table S16), although the optimum Jaccard score for discrimination of flavonoids showed a dependency on the instrument types (Supplementary Table S15, Supplementary Table S3).

Here, we provide the first record of expert knowledge on the fragmentation of a wide range of flavonoid structures, as presented in the supplementary materials of this manuscript. Although these supplementary materials used for the construction of the FsDatabase (Supplementary Tables S2–S8) were calculated and curated manually, the accuracy of flavonoid aglycone identification using this database was an improvement compared to other well-known metabolite prediction tools. These materials provide a useful guide for non-specialists when performing spectral annotation and could be applied to improve model construction in machine learning. The *O*-type substituent list (Supplementary Table S8) could be used for further automation of structural elucidation of modified aglycones such as glycosylated and acylated flavonoids. These materials could guide the prioritization of unmeasured authentic flavonoids for further analysis to expand the coverage of flavonoid compounds, and hence, to enhance the predictive ability of this system because the FsDatabase still included flavonoids without predicted fragment ions. A similar approach using a manual operation could be applied to other natural product groups with relatively rigid backbone structures, including coumarins, lignans, triterpenoids and alkaloids. The expansion of various compounds by construction of the database with a virtual mass fragment to overcome the lack of spectra of authentic compounds will improve the identification rate in metabolomics.

## Methods

### Chemicals

Sources for the standard compounds, which were used for construction of the probable mass fragment database (FsDatabase) and evaluating the FsTool after analysis by a linear ion trap combined with an Orbitrap mass spectrometer (LTQ-Orbitrap, Thermo Fisher Scientific, Waltham, MA), are given in Supplementary Tables S1 and S9, respectively. The following standards for identification of peaks in parsley spectra were purchased from Funakoshi Co. Ltd. (Tokyo, Japan): apigenin, apigenin-7-*O*-glucoside and apigenin-6,8-di-*C*-glucoside.

### Software tools for FsDatabase construction

Xcalibur 2.0.7 (Thermo Fisher Scientific), ChemDraw (Perkin Elmer, Inc., Waltham, MA) and Excel (Microsoft, Redmond, WA) were used for manual operation of the fragment data throughout this study. Calculations of exact masses from chemical formulae were performed using the Formula Calculator tool of PowerGet^27^ based on the masses of atoms and electrons published by IUPAC^28^.

### Data source of the flavonoid structure

For virtual fragment generation, data for 6,867 flavonoid structures were obtained from one of the largest flavonoid databases (metabolomics.jp, http://metabolomics.jp/wiki/Category:FL, previous URL was http://www.metabolome.jp). Data in MDL Molfile format were acquired from the website in 2006. Mismatches between the structures and flavonoid names were manually curated with reference to the original literature cited at the website.

### Analysis of standard compounds for construction of the FsDatabase

We analyzed the standard flavonoid compounds using a linear ion trap (IT) combined with a Fourier transform (FT) ion cyclotron resonance mass spectrometer (LTQ-FT, Thermo Fisher Scientific) because of its ability to provide multistage MS^n^ spectra with high-mass accuracy. The tandem MS^n^ data obtained in electrospray ionization (ESI)-positive mode were used for further analysis. The details of the analytical procedures were described in Supplementary Methods.

### Search tool

A tool for searching the FsDatabase by precursor and fragment masses was developed using the Java Development Kit (JDK 1.6, Oracle Corporation). The candidates were first selected using the precursor *m/z* from the 6,867 flavonoids with a given mass tolerance. The Jaccard index (equation (1)) was used as a similarity score to prioritize the candidates because the intensities of the fragments were not simulated in the FsDatabase.

1 $${\rm{Similarity}}\,{\rm{score}}({\rm{Jaccard}})=\frac{{\rm{Number}}\,{\rm{of}}\,{\rm{query}}\,{\rm{fragments}}\,{\rm{shared}}\,{\rm{with}}\,{\rm{predicted}}\,{\rm{fragments}}}{{\rm{Number}}\,{\rm{of}}\,{\rm{unique}}\,{\rm{fragments}}\,{\rm{in}}\,{\rm{query}}\,{\rm{and}}\,{\rm{predicted}}\,{\rm{fragments}}}$$

The identity of each fragment ion was evaluated using the *m/z* value with a given mass tolerance. The essential fragments (described in the section ‘Development of the fragment prediction rule’ in Supplementary Methods) were used for prioritization of the search results. Candidates with fewer of the essential fragments missing were prioritized higher than those with more of the essential fragments missing at the same similarity score. One of features of this tool is that it does not require selection of adducts, which is usually required for existing tools, because the precursor masses in positive mode are included in the FsDatabase. The tool is available at http://www.kazusa.or.jp/komics/software/FlavonoidSearch.

### Comparison of the prediction accuracy

CFM-ID^14^, FingerID^15^ and MetFrag^16^, which are well-known metabolite prediction tools that use different prediction models, were used for the comparison. The settings for these tools are shown in Supplementary Table S10. The accuracy of each search was compared with the others using the area under the cumulative curve (AUCc) of the frequency ratio of the queries in the efficiency of narrowing down to the correct answer. The details were described in Supplementary Methods.

### Evaluation of the discrimination power

The mass spectra in MassBank^17^ and NIST14 Mass Spectral Library (Ver. June 2014, National Institute of Standards and Technology, Gaithersburg, MD) were used for evaluation of the FlavonoidSearch system for discriminating between flavonoid aglycones and other compounds. The discrimination power was evaluated by binary classification using the Jaccard indices. The result was classed as positive (flavonoid aglycone) or negative (other compounds) when the Jaccard index was greater than zero or zero, respectively. The details of the procedures was described in Supplementary Methods.

### Metabolome analysis of parsley

Semi-comprehensive acquisition of MS^3^ data was performed with a prolonged chromatography separation and data-dependent MS^n^ acquisition. The raw data generated by Xcalibur software were converted to mzXML format using the MSConvert function of ProteoWizard software ver.3.0.6447^29^, and used for peak detection by PowerGet software^27^, which was slightly modified from the original to enable batch processing. At full width at half maximum height, for peaks that eluted between 15 to 90 min and that were annotated as proton adducts, MS^2^ and MS^3^ spectra were extracted from the mzXML file using an in-house Java program. The extracted spectra were used for the FlavonoidSearch. The mass tolerances of FsTool for precursor ion detected in FT-full scan IT-MS^2^ scan and fragment ions in IT-MS^n^ scan were set to 0.01 Da, 0.2 Da and 0.2 Da, respectively. Peaks that were miss detected from background noise signals and those detected also in the analyses of mock samples were removed manually. Peaks in parsley grown in a greenhouse with MS^2^ and MS^3^ data with Jaccard indices >0.3, and which were also detected in parsley grown on a cultivation shelf, were checked manually for the accuracy of MSMS-aglycone annotation from the fragmentation pattern. The most probable types of *O*-substituents attached to each MSMS-aglycone were determined using the accurate masses of neutral loss fragments in MS^2^ data obtained with FT-ICR-MS by referring to the known substituent list (Supplementary Table S8). The novelty of an unknown flavonoid was confirmed using SciFinder (American Chemical Society).

### Hierarchical cluster analysis

Hierarchical cluster analyses of the flavonoids annotated in parsley grown on the cultivation shelf and 15 other plant samples were conducted using semi-comprehensively acquired MS^3^ data. Similarities among the flavonoid aglycone contents were compared based on the frequency of peaks annotated to each symbolized name by the FsTool search. For each symbolized name, a peak frequency was defined as the sum of the *S*_*f*_ values calculated for the search results of all peaks. Each *S*_*f*_ was calculated as 1/*n* for *n* kinds of symbolized names selected with the highest Jaccard indices in a single search. Only search results that showed the highest Jaccard index (>0.3) were used. Hierarchical clustering to produce dendrograms was performed using MeV software (ver. 4.8.1)^30^ with default settings, except for selection of the ‘optimize sample leaf order’ option. For the clustering, the peak frequency values were transformed to log base 10, and missing values were given a value of 1/10^th^ of the minimum value of all the samples. A heat map image was prepared from the log-transformed values using Excel (Microsoft).

### Plant materials

Parsley seeds (*Petroselium crispum*, Paramount) were hydroponically grown in a green house and on a cultivation shelf. Shoots were harvested, frozen in liquid nitrogen, ground to a powder using a mortar and pestle, and stored at −80 °C until use. The details of the growth conditions were described in the Supplementary Methods. The other plant tissues used in this study were from apple fruit (*Malus pumila*), the edible part of broccoli (*Brassica oleracea* var. italica), roots of carrot and purple carrot (*Daucus carota* subsp. sativus), shoots of Chinese cabbage (*Brassica rapa* var. pekinensis), eggplant fruit (*Solanum melongena*), grape flesh (*Vitis* spp.), green pepper and paprika fruit (*Capsicum annuum* ‘grossum’), peeled fruit of mandarin oranges (*Citrus unshiu*) and oranges (*Citrus sinensis*), the edible part of onions (*Allium cepa*), shoots of spinach (*Spinacia oleracea*), squash fruit (*Cucurbita maxima*) and strawberry fruit (*Fragaria* x *ananasa* Duchesne ex Rozier). Samples of these vegetables and fruits were purchased at a local market in Tochigi prefecture, Japan. For each vegetable or fruit, three individual samples were purchased, freeze-dried, and then ground to powder using a mortar and pestle. Then, the three powdered samples for each fruit or vegetable were mixed together and stored at −80 °C until use.

### LC-MS analyses

Plant samples were extracted with methanol (final concentration 75% *v/v*, containing 25 µM of 7-hydroxy-5-methylflavone as an internal standard). After homogenizing the samples, centrifugation, and filtration, hydrophobic compounds in the filtrate were removed by absorption to a C18 silica column (MonoSpin C18, GL Science, Tokyo, Japan). Standard compounds for FsDatabase construction and identification of parsley peaks were dissolved in methanol. LC-MS analyses were performed using Agilent 1100 or 1200 systems (Agilent, Palo Alto, CA) coupled to a Finnigan LTQ-FT (Thermo Fisher Scientific) or a Finnigan LTQ-Orbitrap XL (Thermo Fisher Scientific). The binary raw data from Xcalibur (.raw) and their experimental metadata for plant samples are deposited at MassBase^27^ and Metabolonote^31^, respectively. Their IDs and peak data are available at the KOMICS website^27^ (http://www.kazusa.or.jp/komics/software/FlavonoidSearch). The details of the extraction and analysis procedures were described in Supplementary Methods.

### Other methods

The details of the other methods were described in Supplementary Methods that includes follows: Assignment of information to fragment ions; Development of the MSMS-category rule; Development of index of hydrogen deficiency (IHD) heuristics; Development of the fragment prediction rule; Construction of the FsDatabase and substituent table; and Data for evaluation of the tool.

## Electronic supplementary material

Supplementary Information

Supplementary Table S1

Supplementary Table S2

Supplementary Table S3

Supplementary Table S5

Supplementary Table S6

Supplementary Table S7

Supplementary Table S8

Supplementary Table S9

Supplementary Table S16

Supplementary Table S17

Supplementary Table S19
