# Supplementary material for: FlavonoidSearch: A system for comprehensive flavonoid annotation by mass spectrometry
Source: Sci Rep. 2017 Apr 28;7:1243. doi: 10.1038/s41598-017-01390-3 (PMC5430893; doi:10.1038/s41598-017-01390-3)
Supplement: Supplementary file 1 — Supplementary Information [file 41598_2017_1390_MOESM1_ESM.pdf]

Supplementary Information

FlavonoidSearch: A system for comprehensive flavonoid annotation by mass spectrometry

Nayumi Akimoto, Takeshi Ara, Daisuke Nakajima, Kunihiro Suda, Chiaki Ikeda, Shingo Takahashi, Reiko Muneto, Manabu Yamada, Hideyuki Suzuki, Daisuke Shibata, Nozomu Sakurai

---

Contents

Supplementary Methods **page 4**

Supplementary Figure S1. Comparison of the accuracy of the search tools. **page 14**

Supplementary Figure S2. Frequency distribution of spectra with various Jaccard indices from FlavonoidSearch. **page 18**

Supplementary Figure S3. Receiver operator characteristic (ROC) curves for the discrimination of flavonoid aglycones. **page 20**

Supplementary Figure S4. Flavonoids in parsley samples (an overall view of Figure 4a). **page 22**

Supplementary Table S1. Standard compounds used to obtain MS^n spectra for construction of the probable mass fragment database (FsDatabase). **Excel file**

Supplementary Table S2. Assignment of formulae and structural information to the fragment ions observed in the measurements of standards. **Excel file**

Supplementary Table S3. The MSMS-category rule. **Excel file**

Supplementary Table S4. Trends in the intensities of the fragment ions. **page 23**

Supplementary Table S5. Characteristic fragment ions observed in each MSMS-category. **Excel file**

Supplementary Table S6. The fragment prediction rule. **Excel file**

Supplementary Table S7. The predicted mass fragment database (FsDatabase) **Excel file**

Supplementary Table S8. The *O*-type substituents. **Excel file**

Supplementary Table S9. Standard compounds analyzed using a linear ion trap combined with an Orbitrap mass spectrometer (LTQ-Orbitrap) for evaluation of the FlavonoidSearch system. **Excel file**

Supplementary Table S10. Parameter settings for the search tools used in the evaluation of accuracy and flavonoid discrimination power. **page 24**

Supplementary Table S11. Number of query spectra and mean number of candidate molecules in the search results for each dataset. **page 25**

Supplementary Table S12. Areas under the cumulative curves in the accuracy estimation. **page 26**

Supplementary Table S13. Statistics for discrimination of flavonoid aglycones using the Jaccard index from FlavonoidSearch. **page 27**

Supplementary Table S14. False negatives and their predicted fragments in the FlavonoidSearch database. **page 28**

Supplementary Table S15. Maximum accuracy and Youden index for the discrimination of flavonoid aglycones using the Jaccard index from FlavonoidSearch. **page 29**

Supplementary Table S16. Instruments used in the evaluation. **Excel file**

Supplementary Table S17. Annotated flavonoids in parsley. **Excel file**

Supplementary Table S18. Combination of *O*-substituents and MSMS-aglycones detected in parsley. **page 30**

Supplementary Table S19. Peak frequencies for the flavonoids in various vegetables and fruits. **Excel file**

References **page 31**

Supplementary Methods

Analysis of standard compounds for construction of the FsDatabase

We analyzed 139 standard flavonoid compounds to construct a mass spectral dataset of various types of flavonoids measured using the same instrumental conditions. This dataset was used to confirm the existing knowledge of fragmentation, and to investigate relationships between the structures and fragmentation patterns. We purchased as many standard compounds as we could from major chemical vendors in Japan, including Funakoshi Co., Ltd. (Tokyo, Japan), Sigma-Aldrich (St. Louis, MO) and Nacalai Tesque, Inc. (Kyoto, Japan) in 2006 (**Supplementary Table S1**). As we decided to develop the fragmentation rule for flavonoid classes (MSMS-categories) which have more than two standard measurements, additional standard compounds were purchased from Plantech UK (Berkshire, UK) to expand the coverage of MSMS-categories. We analyzed these standards using a linear ion trap (IT) combined with a Fourier transform (FT) ion cyclotron resonance mass spectrometer (LTQ-FT, Thermo Fisher Scientific) because of its ability to provide multistage MS^n spectra with high-mass accuracy. These spectra could be used for precise assignment of formulae and structural information to the fragments. Only the tandem MS^n data obtained in electrospray ionization (ESI)-positive mode were used for further analysis because this mode provided more characteristic fragmentation than ESI-negative mode [1]. Essentially the same fragmentation was observed in MS^2 of an MSMS-aglycone and MS^3 of its *O*-glycoside, and the MSMS-aglycone–based prediction of mass fragments could be applied to MS^n data.

Assignment of information to fragment ions

Assignment of chemical formulae and structural information to the observed fragment ions was carried out manually. This resulted in 1,080 assignments from FT-MS^2 and 1,109 from IT-MS^2 data, which were compiled in Excel spread sheets (**Supplementary Table S2**). The IT-MS^2 data were used to assign fragments because they showed high sensitivity. A website, MS-MS Fragment Viewer (http://webs2.kazusa.or.jp/msmsfragmentviewer/), for publication of the results was developed using Perl 5.8.8 and Java Development Kit (JDK 1.6, Oracle Corporation, Redwood City, CA) and is running on a Red Hat Enterprise Linux Server 7.1 with Apache 2.4.6.

Development of the MSMS-category rule

Characteristic fragmentation occurred in each flavonoid class [2-20]. However, we found

that when flavonoids were classified according to their backbone, different fragmentation patterns sometimes occurred in the same class. For example, in the chalcone class, different mass fragments were observed for the compounds with and without a 2′-hydroxyl group (**Supplementary Table S2**). This difference was likely caused by the 2′-hydroxyl group, which might generate an intermediate structure the same as an isomeric flavanone [14]. Because of the differing fragmentation within classes, a new classification system was required to distinguish structures using their fragmentation patterns. Therefore, we developed the MSMS-category classification system, which is based on the structural features shown in **Supplementary Table S3**. In addition to the traditional flavonoid classification by the backbone structure, the flavonoid structures were further sub-divided using the types and positions of substituents that could affect C-ring cleavage. We considered substituents, hydroxyl and oxo groups and other *C*-type substituents, that were bonded to the propane moiety and found in the standards we analyzed. Because the effects of methoxyl group and *O*-type substituents on the fragmentation patterns were not clear from the results for the standards, we replaced them with hydroxyl groups. Using this classification system, the 101 MSMS-aglycone standards (**Supplementary Table S1**) were classified into 16 MSMS-categories (**Supplementary Table S4**).

Index of hydrogen deficiency (IHD) heuristics

We found that trends in the intensities of the fragment ions were related to the IHD of the backbone structure and combinations of the substituents. The trends in the intensities of the fragment ions were manually extracted from the data in **Supplementary Table S2**. In addition to the fragment ions derived from cleavage of the propane moiety (C-ring) of the C_6-C_3-C_6 backbone structure and neutral losses of small substituents [21], we checked those derived from neutral losses of the substituents bonded to the A- and/or B-rings (**Supplementary Table S4**). Intensities for the fragment ions in the MSMS-categories are represented as three intensity levels (++, + and –, see the footnote to **Supplementary Table S4**). We observed the following general trends, which we refer to as 'IHD heuristics', and these could be used to develop the fragment prediction rule (see next section):

1) The intensity levels for fragment ions derived from C-ring cleavage and small neutral loss showed similar trends for compounds from MSMS-categories with the same IHD.

2) Fragments derived from C-ring were more intense for compounds in MSMS-categories with lower IHD than those with higher IHD. By contrast, fragments derived from small neutral loss and neutral loss of substituents on the A-/B-rings were

more intense for compounds in MSMS-categories with higher IHD than lower IHD.
3) For a particular IHD, the intensity levels for fragments derived from neutral loss of substituents on the A-/B-rings differed depending on where they were derived from.
4) Fragments derived from C-ring cleavage and small neutral loss were not affected by the presence of a methoxyl group on the A-/B-rings. Therefore, we assumed that each substituent on the A-/B-rings could have both an independent and combined effect on fragmentation.

The IHD was calculated based on the number of carbon (*a*) and hydrogen (*b*) atoms in a positively charged backbone structure ($C_aH_bO_c^+$) according to the following **equation (2)**:

$$(2a + 2 - b) / 2. \qquad (2)$$

Development of the fragment prediction rule

To ensure reliable prediction of fragments, we selected 6,102 out of 6,867 flavonoids in 14 MSMS-categories that had more than two standard measurements as prediction targets. Among these 6,102 flavonoids, we selected 4,843 that had substituents on the A- and B-rings that were the same as those found in the measured standards. Among these 4,843 flavonoids, we selected 3,574 with particular combinations of MSMS-aglycones and the substituents, and that had at least one standard measurement. Then, using the IHD heuristics discussed above, we added flavonoids that had previously unmeasured combinations of the 14 MSMS-categories and substituents on the A- or B-rings as prediction targets. This included dihydroflavonol combined with hydroxyl and methoxyl groups, pterocarpan with hydroxyl groups, flavan-3-ol with hydroxyl and methoxyl groups, flavonoids with IHD > 9.5 and prenyl or [2,3:m,n]-(6,6-dimethylpyrano) groups and flavonoids with IHD > 10.5 and *C*-glucosyl groups. In total, 4,119 flavonoids were targeted for fragment prediction. Characteristic fragment ions that were expected to be reproducibly observed in each MSMS-category with the A-/B-ring substituents were manually compiled (**Supplementary Table S6**) with reference to the measurement results (**Supplementary Table S5**). Because gallate esterified at the 3-C position in flavan 3-ol affected the fragmentation pattern, the fragment ions related to gallate and its analogues esterified at the 3-C position in flavan 3-ol were added to the table (**Supplementary Table S6**). The results in this table are used for the 'Fragment prediction rule'. Once an MSMS-category is determined from the structure using the MSMS-aglycone rule and the MSMS-category rule, the possible

mass fragments are predicted using the table. The observed fragments were assigned as follows: 56 fragments from small neutral loss, 10 A-ring-derived fragments from C-ring cleavage and 13 B-ring-derived fragments from C-ring cleavage. Fragments that will most likely be observed, because they were observed in every measurement of the standard compounds, are referred to as 'Essential fragments'.

Construction of the FsDatabase and substituent table

The MSMS-aglycone rule and the MSMS-category rule were applied to the 6,867 flavonoids, resulting in 3,678 unique MSMS-aglycones and 124 MSMS-categories. The fragment prediction rule was applied to the selected flavonoids as described above, and we obtained possible fragments for 4,119 flavonoids, including 1,489 MSMS-aglycones in 14 MSMS-categories (referred to as the FsDatabase, **Supplementary Table S7**). The precursor ion for a flavonoid were assumed to be $[M+H]^+$ or $[M]^+$ according to the MSMS-aglycone. Most of the MSMS-aglycone names include common names. To facilitate understanding of the structural similarities among the flavonoids, we created 'symbolized names' for them. The symbolized names include information about the MSMS-category, types and number of substituents and some other structural information. The 419 unique *O*-type substituents attached to the MSMS-aglycones were manually compiled (**Supplementary Table S8**).

Data for evaluation of the tool

The MS/MS data from 101 standard MSMS-aglycones used in construction of the FsDatabase, including IT and FT-ICR data measured by LTQ-FT, were used to evaluate the tool. MS/MS spectra of 87 standards were measured using a LTQ-Orbitrap (**Supplementary Table S9**) to provide data from another instrument for comparison. Data in the MassBank database [22] were downloaded in November 21, 2014, and data that matched all the following conditions were selected: AC$INSTRUMENT_TYPE starts with LC-ESI; AC$MASS_SPECTROMETRY: MS_TYPE is MS2; AC$MASS_SPECTROMETRY: ION_MODE is POSITIVE; MS$FOCUSED_ION: PRECURSOR_M/Z has a value; MS$FOCUSED_ION: PRECURSOR_TYPE is [M+H]+, $[M]^{*+}$, $[M]^{+*}$, $[M]^+$ or M^+; and CH$FORMULA contains a valid formula. The records with LC-ESI-ITFT or LC-ESI-QTOF as AC$INSTRUMENT_TYPE field were included in the selected MassBank data whose CH$LINK CAS field has a CAS identifier as one of the 6,867 flavonoids. NIST14 Mass Spectral Library (Ver. June 2014, National Institute of Standards and Technology, Gaithersburg, MD) was purchased from the Japan Association for International Chemical Information (Tokyo, Japan). In the MS/MS

Library of small molecules, data that matched all the following conditions were selected: spectrum type was MS2; ion mode was P; ionization technique was ESI; precursor type was $[M+H]^+$ or $[M]^+$.; a valid formula was given; and a InChIKey was given. The records with Q-TOF, IT-FT/ion trap with FTMS and IT/ion trap, HCD or QqQ as Instrument type were included in the selected NIST14 data whose InChIKey skeleton (see next section) was included in the 6,867 flavonoids. The most intense 20 ions of the MS^2 scan were used for the evaluation. The precursor masses for the MassBank and NIST records were calculated from the formulae because some records contained incorrect values. Spectra containing more than two fragments excluding the precursor ion were used. When evaluating instrument dependency, the MassBank records with AC$INSTRUMENT_TYPE of LC-ESI-ITFT and LC-ESI-QTOF were categorized as ion trap/Fourier transform ion cyclotron MS (ITFT) and quadrupole time–of–flight MS (QTOF), respectively. NIST record with Instrument type Q-TOF, IT-FT/ion trap with FTMS and IT/ion trap, HCD and QqQ were categorized as quadrupole time-of-flight MS (QTOF), ion trap/Fourier transform MS (ITFT), higher–energy collisional dissociation MS (HCD) and triple quadrupole MS (QqQ), respectively. The instrument types included in MassBank and NIST are shown in **Supplementary Table S16**.

Comparison of the prediction accuracy

CFM-ID [23], FingerID [24] and MetFrag [25], which are well-known metabolite prediction tools that use different prediction models, were used for the comparison. The settings for these tools are shown in **Supplementary Table S10**. The mass tolerances for the precursor and fragment ions were determined by calculating the differences between the measured and theoretical values. MS/MS tolerances for each instrument type in MassBank and NIST were estimated using the precursor ions detected in the MS/MS spectra in the same manner. The Kyoto Encyclopedia of Genes and Genomes (KEGG) database was selected as the common target database for each tool. Structure Data Format (SDF) files for the standards were used for the local SDF search by MetFrag. The MetFrag search was performed using the Java tool MetFragCommanLineTool.jar, which was downloaded from the MetFragCL on github, according to the provided instructions. A CFM-ID search was performed using CFM-ID 2.0 windows binary and a pre-trained metab_se_cfm dataset available at the website of the tool. Both Jaccard indices and dot-product similarity values were calculated. A FingerID search was performed using the FingerID 1.4 Phyton script. Data from QTOF in MassBank, and QTOF, HCD and QqQ in NIST were calculated with pre-trained LC-ESI-QTOF data, and the other data were calculated with pre-trained LC-ESI-ITFT-CID data.

Calculations with MetFrag and CFM-ID were carried out on a PC with the Windows 7 (Microsoft, 64 bit) OS, Intel Core i7-2600 3.4 GHz CPU and 8 GB RAM. Calculations with Finger ID were carried out with Python 2.7 on the CentOS7 guest OS in a virtual environment (VirtualBox 5.0.12, Oracle Corporation) running on the same Windows 7 PC.

The hash version (InChIKey) of the IUPAC International Chemical Identifier (InChI) was calculated using Chemistry Development Kit (CDK, 1.4.19) [26] from MDL Mol files for standards flavonoids, NIST data and KEGG data downloaded on August 15, 2015, and from InChI codes of MassBank data. The first block of InChIKey (referred to as the 'InChIKey skeleton'), which represents the connectivity of the atoms, ignoring the stereochemistry, was used for matching compounds among the different types of data, including standard compounds, KEGG IDs, MassBank records and NIST records.

Because candidate numbers and their prioritization in the search results differed with the tools and queries, the accuracy of each search was compared with the others using the area under the cumulative curve (AUCc), which was calculated as follows. First, the efficiency of narrowing down to the correct answer (E_f; **equation (3)**) was calculated for each query.

$$E_f = O_{\text{correct}} / \text{Number of all candidates}, \tag{3}$$

where O_{correct} is the number of candidates with a similarity value higher than or equal to the correct answer. Next, a ratio of queries (R_q) value was calculated as follows:

$$R_q = \text{Number of queries in a range of } E_f / \text{Number of all queries}. \tag{4}$$

Curves were constructed for the cumulative R_q for different E_f ranges between 0 and 1 with an interval of 0.1 (**Fig. 3a** and **Supplementary Fig. S1**). The cumulative curve will move closer to the upper left-hand corner of the figure when highly narrowed-down results are obtained for a high number of queries. Therefore, the AUCc can be used to evaluate the search accuracy (**Supplementary Table S12**).

Because the FsDatabase contains many more flavonoids than in the KEGG database, the number of candidates from the FsDatabase was larger than that from the KEGG database (**Supplementary Table S11**). To exclude the effect of the differences in the candidate numbers from the accuracy estimation, we prepared a KEGG-mimicked FsTool results as follows: from the original FsTool results, flavonoids which were not registered in KEGG were removed, and the other compound entries in KEGG that had

the same mass as the precursor ion used as the query were added. Because there were still slight differences in the candidate numbers between the tools, probably because of differences among the KEGG database versions used in each tool, we adjusted the denominator in the E_f calculation (**equation (3)**) to the average value of those from other tools (FlavonoidSearch (KEGG) in **Fig. 3a, Supplementary Fig. S1** and **Supplementary Tables S11** and **S12**). Supposing that a very limited number of flavonoids are registered in the KEGG database, and that many other isobaric compounds exist, the calculation could give artificially high accuracy if the flavonoid aglycones are not selected using FlavonoidSearch. Therefore we performed a simulation assuming that all flavonoids among the candidates were correct regardless of their Jaccard index (FlavonoidSearch (KEGG Sim) in **Supplementary Fig. S1** and **Supplementary Table S12**). For further comparison, we conducted an evaluation using only the queries whose correct results were found by all tools (Common compounds in **Supplementary Fig. S1**, and **Supplementary Tables S11** and **S12**). Results obtained with common compounds are shown in **Fig. 3a**.

Evaluation of the discrimination power

The flavonoid aglycones from MassBank and NIST data were selected as follows. First, records with InChIKey skeletons of flavonoid aglycones were selected as a whitelist. Next, filters were applied to select compounds with names containing 'flavon', 'flavan', 'chalcon' or 'catechin', and compounds having FsTool scores greater than zero. These compounds were designated as potential candidates. Then, the structures of the potential candidates were checked manually and only those with an aglycone structure were added to the whitelist. The power of FsTool for discriminating flavonoid aglycones from other compounds was evaluated by binary classification using the Jaccard indices. The result was classed as positive (flavonoid aglycone) or negative (other compounds) when the Jaccard index was greater than zero or zero, respectively. The discrimination power was first evaluated for all records (**Supplementary Table S13**). Because true negative was too large, evaluation of maximum discrimination power was performed excluding records with Jaccard indices of zero (**Supplementary Table S15, Figs. 3b** and **3c** and **Supplementary Fig. S2**). MassBank and NIST databases included multiple records measured for the same compound. To remove bias from the repetitive measurements, unique structures were extracted from the whitelist based on the InChIKey skeleton ('Unique structures' in **Supplementary Figs. S2** and **S3** and **Supplementary Tables S13–S15**). In this case, the average of the Jaccard indices was used for evaluation of the discrimination power. Results from unique structures are

shown in **Figs. 3b and 3c**. Because the variation in fragmentation with HCD is larger than that with collision-induced dissociation (CID), the improvement of the area under the curve (AUC) in the HCD data from NIST for the unique structures implied that many false negative records were included despite the existence of predicted fragments (**Supplementary Table S14**).

Growth conditions of parsley

Parsley seeds (*Petroselium crispum*, Paramount) were purchased from Kaneko Seeds Col. Ltd. (KS100-542, Gunma, Japan). The seeds were germinated on wet filter paper (Whatman 1002-150, GE Healthcare Japan) in a petri dish. The seedlings were grown for 14 days on a cultivation shelf under fluorescent lamps (Biolux-A, NEC Corp., Tokyo, Japan) using a 16-h light/8-h dark cycle at 25 °C. The seedlings were transplanted into vermiculite (10865595 SK Agri K.K., Gunma, Japan), and grown for 164 days under the same conditions as above with fertilization with a culture medium containing 3 mM KNO_3, 1 mM $Ca(NO_3)_2$, 0.5 mM $MgSO_4$, 0.5 mM $NH_4H_2PO_4$, 50 µM Fe-EDTA, 49 µM H_3BO_3, 9 µM $MnSO_4$, 0.8 µM $ZnSO_4$, 0.3 µM $CuSO_4$ and 0.1 µM Na_2MoO_4. Parsley seeds were also germinated on wet sand. After 20 days, the seedlings were transplanted into baked clay pebbles (Hydroball, Toshi Engei Co., LTD, Saitama, Japan) in a green house, and grown for 63 days with the same culture medium as above.

Metabolite extraction from the plant samples

Frozen parsley powder (300 mg) was extracted with 900 µL of methanol containing 25 µM of 7-hydroxy-5-methylflavone as an internal standard. For the other vegetables and fruits, a freeze-dried sample (30 mg) was extracted with 1,200 µL of 75% *v/v* methanol containing the same concentration of the internal standard. After homogenizing the samples by mixing twice for 2 min each time using a Mixer Mill MM 300 (QIAGEN K.K., Tokyo, Japan) at 25 Hz, the homogenates were centrifuged (17,400 ×*g* for 5 min at 4 °C). After recovery of the supernatant from each homogenate, it was filtered through a 0.2-µm polytetrafluoroethylene (PTFE) membrane (Millipore). Hydrophobic compounds in the filtrate were removed by absorption to a C18 silica column (MonoSpin C18, GL Science, Tokyo, Japan). The eluate was used for LC-FT-ICR-MS analysis. Mock samples were prepared as above without adding the plant materials.

Preparation of standard compounds for peak identification in parsley

Standard compounds for identification of parsley peaks were dissolved in methanol (final concentration 0.1 mM), and filtered through 0.2 µm PTFE membranes (Millipore).

An aliquot (20 μL) of each solution was analyzed by the same procedure used for parsley analysis (described below).

LC-MS analyses

Standards for the FsDatabase construction were analyzed using an Agilent 1100 system (Agilent, Palo Alto, CA) coupled to a Finnigan LTQ-FT (Thermo Fisher Scientific). An aliquot (5–20 μL) of a methanol solution (25–100 μg/mL) was applied to a TSK-gel column ODS-100Z (4.6 × 250 mm, 5 μm; TOSOH Corporation, Tokyo, Japan). Water (HPLC grade; solvent A) and acetonitrile (HPLC grade; solvent B) were used as the mobile phase with 0.1% *v/v* formic acid added to both solvents. The gradient program was as follows: 3% B (0 min), 30% B (25 min), 90% B (40 min), 90% B (45 min), 95% B (45.1 min), 95% B (50 min), 3% B (50.1 min) and 3% B (57 min). The flow rate was set to 0.5 mL/min, and the column oven temperature was set at 30 °C. Compounds were detected in ESI-positive mode over the *m/z* range 150–1000. Multistage MS^n analyses were carried out using collision-induced dissociation in a linear ion trap detector with a normalized collision energy of 35.0% and an isolation width of 4.0 (*m/z*). Both an ion trap detector and FT-ICR detector were used at a mass resolution of 25,000 (at *m/z* 400). The ESI settings were a spray voltage of 4.0 kV and capillary temperature of 300 °C. The nitrogen sheath gas and auxiliary gas were set at 40 and 15 arbitrary units, respectively. To monitor the HPLC eluate, a photodiode array detector was used with a wavelength range of 200–650 nm. Data were acquired and browsed using Xcalibur software version 2.0.7 (Thermo Fisher Scientific).

For evaluation of the accuracy of FsTool, standards were analyzed using an Agilent 1200 system (Agilent) coupled to a Finnigan LTQ-Orbitrap XL (Thermo Fisher). LC separation and mass analysis were performed as described above with the following modifications. Five microliters of the 75% methanol solution (10 μg/mL) was applied to a TSK-gel column ODS-100V (3 × 50 mm, 5 μm; TOSOH). The gradient program was as follows: 3% B (0 min), 97% B (15 min), 97% B (20 min), 3% B (20.1 min) and 3% B (25 min). MS^n analyses in collision-induced dissociation were carried out in a linear ion trap detector. The column oven temperature was set to 40 °C. The mass range was *m/z* 100–1500.

The other plant samples were analyzed using an Agilent 1100 system (Agilent) coupled to a Finnigan LTQ-FT (Thermo Fisher Scientific). LC separation and mass analysis were performed as described above for FsDatabase construction, with the following modifications. Twenty microliters of extract was applied to the TSK-gel column ODS-100V (4.6 × 250 mm, 5 μm; TOSOH). The gradient program was as follows:

3% B (0 min), 97% B (90 min), 97% B (100 min), 3% B (100.1 min) and 3% B (107 min). The flow rate was set to 0.25 mL/min (0–100 min) and 0.5 mL/min (100.1–107 min). The column oven temperature was set to 40 °C. The mass range was *m/z* 100–1500. The following three methods for MS^n analysis were performed for parsley: 1) full mass scan with FT-ICR at a resolution of 100,000, and MS^2 scans for the most intense five ions of the full mass scan with ion trap (IT); 2) full mass scan with FT-ICR at a resolution of 12,500, MS^2 scans for the most intense five ions of the full mass scan with IT, and MS^3 scans for the most intense two ions of the MS^2 scan with IT; and 3) full mass scan with FT-ICR at a resolution of 12,500, and MS^2 scan for the most intense five ions of the full mass scan with FT-ICR. A dynamic exclusion setting was applied for methods 1) and 2) as follows: repeat count, three; repeat duration, 30 s; exclusion list size, 500; margin, 10 ppm; and exclusion duration, 20. Only method 2) was applied to analysis of the other plant samples. The binary raw data from Xcalibur (.raw) and their experimental metadata for plant samples are deposited at MassBase [27] and Metabolonote [28], respectively. Their IDs and peak data are available at the KOMICS website [27] (http://webs2.kazusa.or.jp/komics/software/FlavonoidSearch).

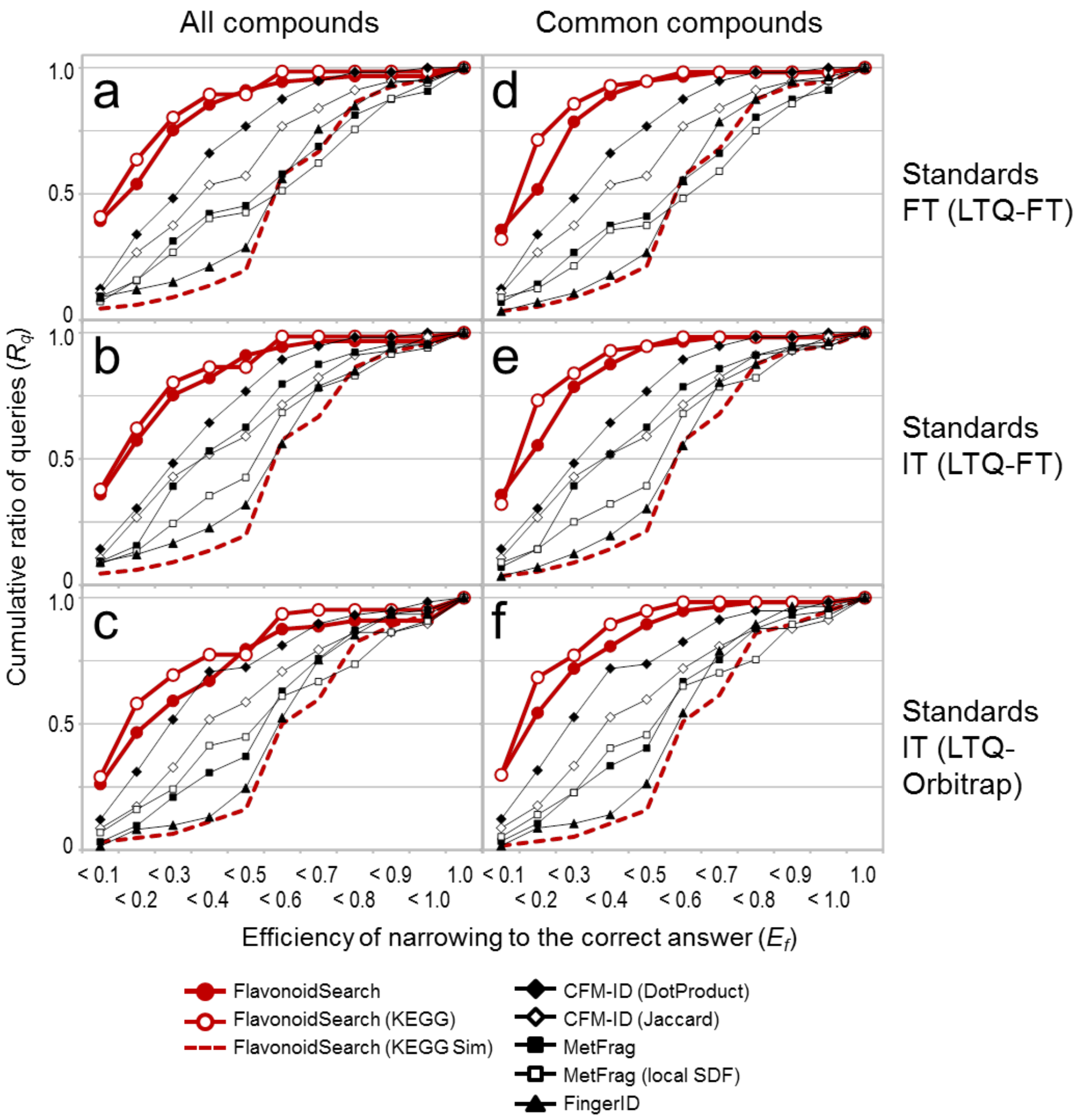

Supplementary Figure S1. Comparison of the accuracy of the search tools. The accuracy of each search tool was evaluated using the area under the cumulative curve (AUCc) for a plot of the cumulative ratio of queries (*Y*-axis) to the efficiency of narrowing down to the correct answer (*X*-axis) (see **Supplementary Methods** for definitions and equations for these parameters). A high AUCcs is indicative of high accuracy. Results are shown for standard flavonoids measured using the following mass spectrometry (MS) techniques: 1) in-house data includes Fourier transform (FT) data from a linear ion trap combined with a FT ion cyclotron resonance mass spectrometer (LTQ-FT) (**a** and **d**), ion trap (IT) data from the LTQ-FT (**b** and **e**) and IT data from a linear ion trap combined with an Orbitrap mass spectrometer (LTQ-Orbitrap, **c** and **f**); 2) MassBank data includes that from both IT/FT MS (ITFT) and quadrupole time-of-flight MS (QTOF) plotted together (**g** and **j**), and separated into ITFT (**h** and **k**) and QTOF (**i** and **l**); and 3) NIST14 data includes that from multiple MS techniques plotted together (**m** and **r**), and separated into ITFT (**n** and **s**), QTOF (**o** and **t**), triple quadrupole MS (QqQ) (**p** and **u**) and higher-energy collisional dissociation MS (HCD) (**q** and **v**). For a fair comparison, the search space of

FlavonoidSearch was adjusted to the same as that of the KEGG database, which was used for all the other tools except the MetFrag search using local Structure Data Format (SDF) files. The adjusted search results are designated as FlavonoidSearch (KEGG). Results from a simulation without prioritization of the correct answer for the KEGG flavonoids are designated as FlavonoidSearch (KEGG Sim). The simulated results were used to evaluate false positives which might cause by the low number of flavonoids and the high number of other compounds in the KEGG database. The figures are separated into two columns, with the 'All compounds' column showing results that were obtained using the data from all available compounds (**a**–**c**, **g**–**i** and **m**–**q)**. The other column ('Common compounds') shows results from only those compounds that were searchable by all tools (**d**–**f**, **j**–**l** and **r**–**v**). The 'Common compounds' figures allow for a fair comparison of the accuracy of the search tools.

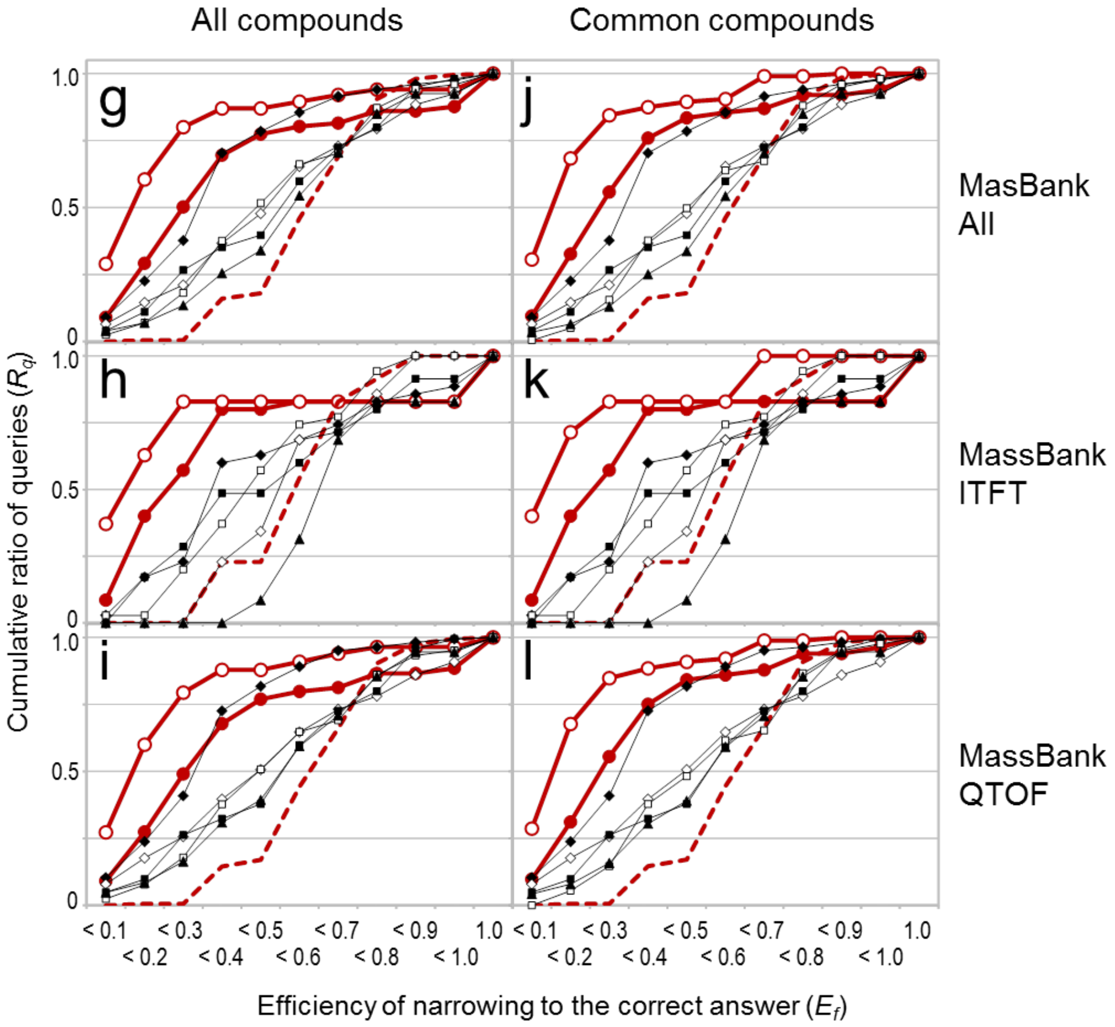

Supplementary Figure S1. Comparison of the accuracy of the search tools. ***Continued.***

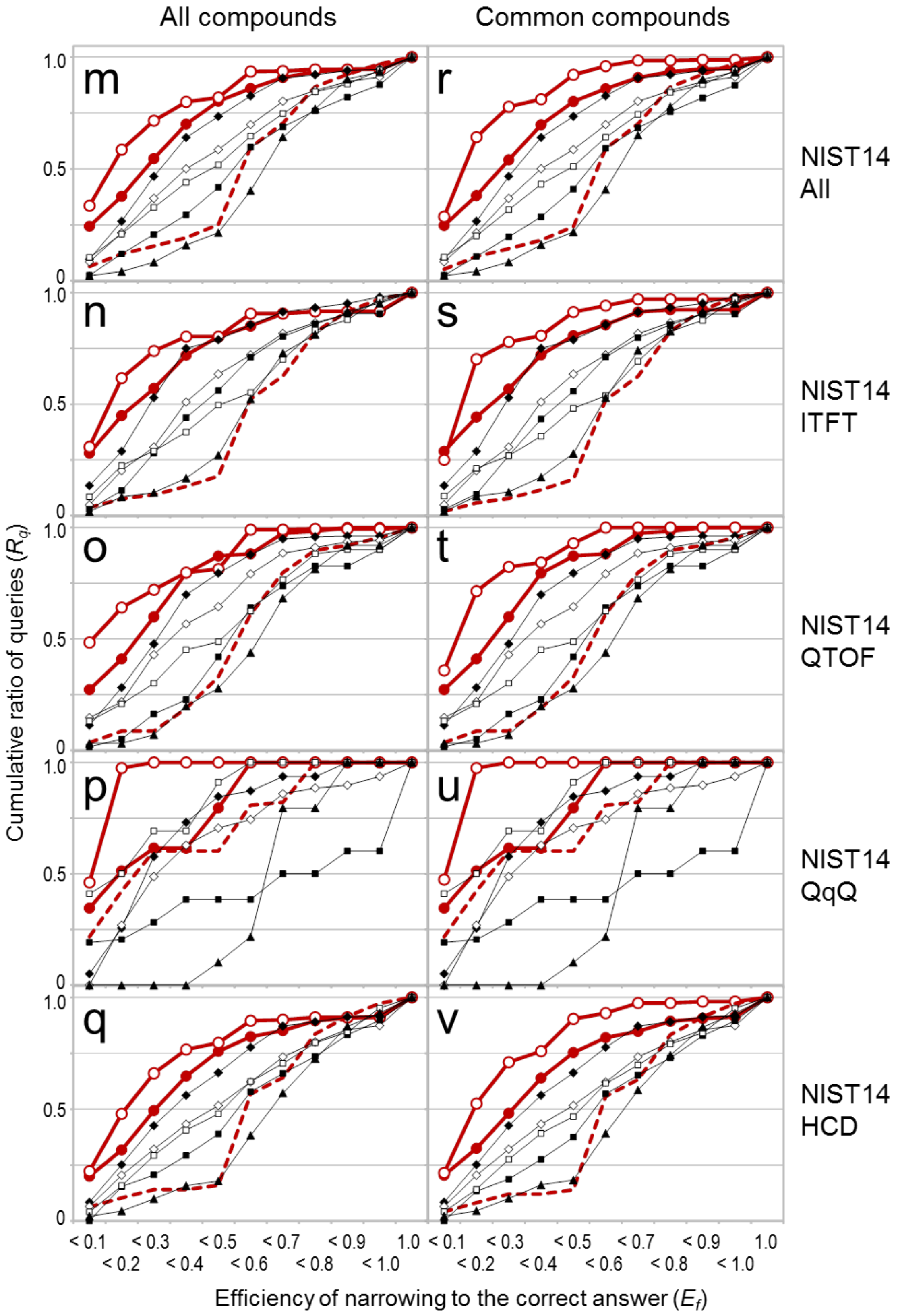

Supplementary Figure S1. Comparison of the accuracy of the search tools. *Continued.*

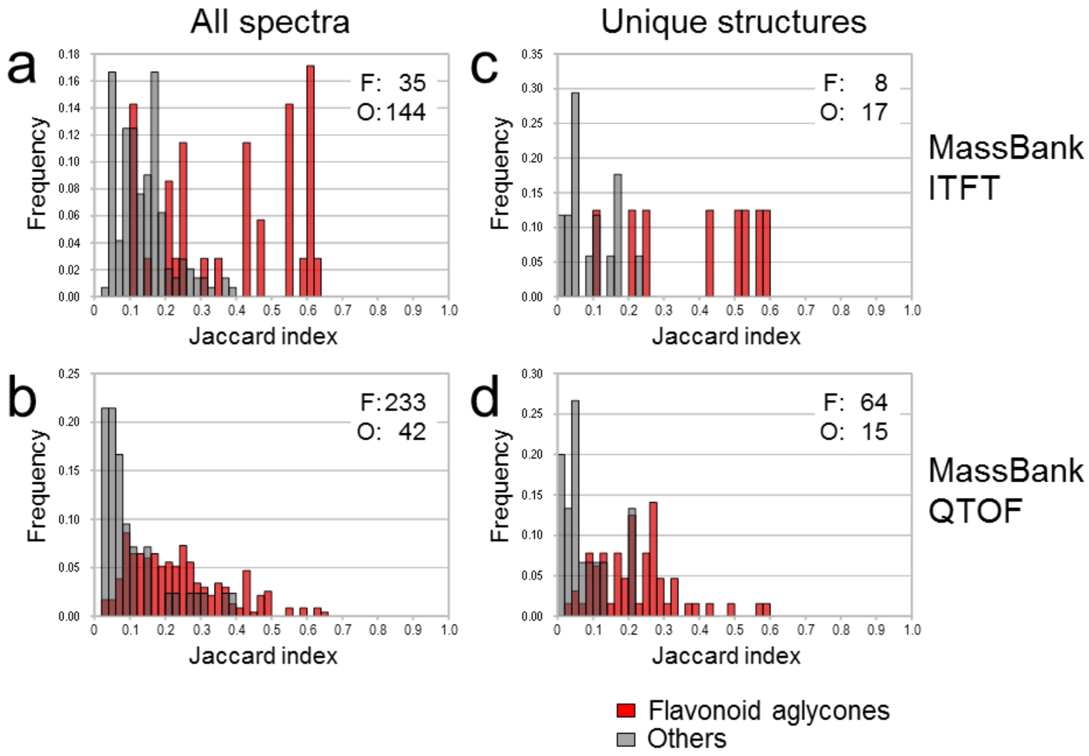

Supplementary Figure S2. Frequency distribution of spectra with various Jaccard indices from FlavonoidSearch. Spectra of flavonoid aglycones (red bars) and other compounds (gray bars) were searched using FsTool for FlavonoidSearch. The results show the frequency of spectra with a Jaccard index within a particular range for the most likely candidate flavonoid selected by FsTool. The frequency is the ratio of the records with the range of Jaccard index to all records in each compound group. Only the records with Jaccard indices greater than zero were used for the ratio calculation. The numbers of spectra for flavonoid aglycones (F) and other compounds (O) are given in the top right hand corner of each figure. To remove bias from repeat measurements of the same compound, unique structures were extracted using the first block of InChiKey, and these results are shown in the 'Unique structures' column (**c**–**d** and **i**–**l**). The average Jaccard index for the repeat measurements was used for the calculation. Data were obtained from MassBank (ion trap/Fourier transform MS (ITFT), **a** and **c**; and quadrupole time-of-flight MS (QTOF), **b** and **d**) and NIST14 (IT/FT (ITFT), **e** and **i**; QTOF, **f** and **j**; triple quadrupole MS (QqQ), **g** and **k**; and higher-energy collisional dissociation MS (HCD), **h** and **l**).

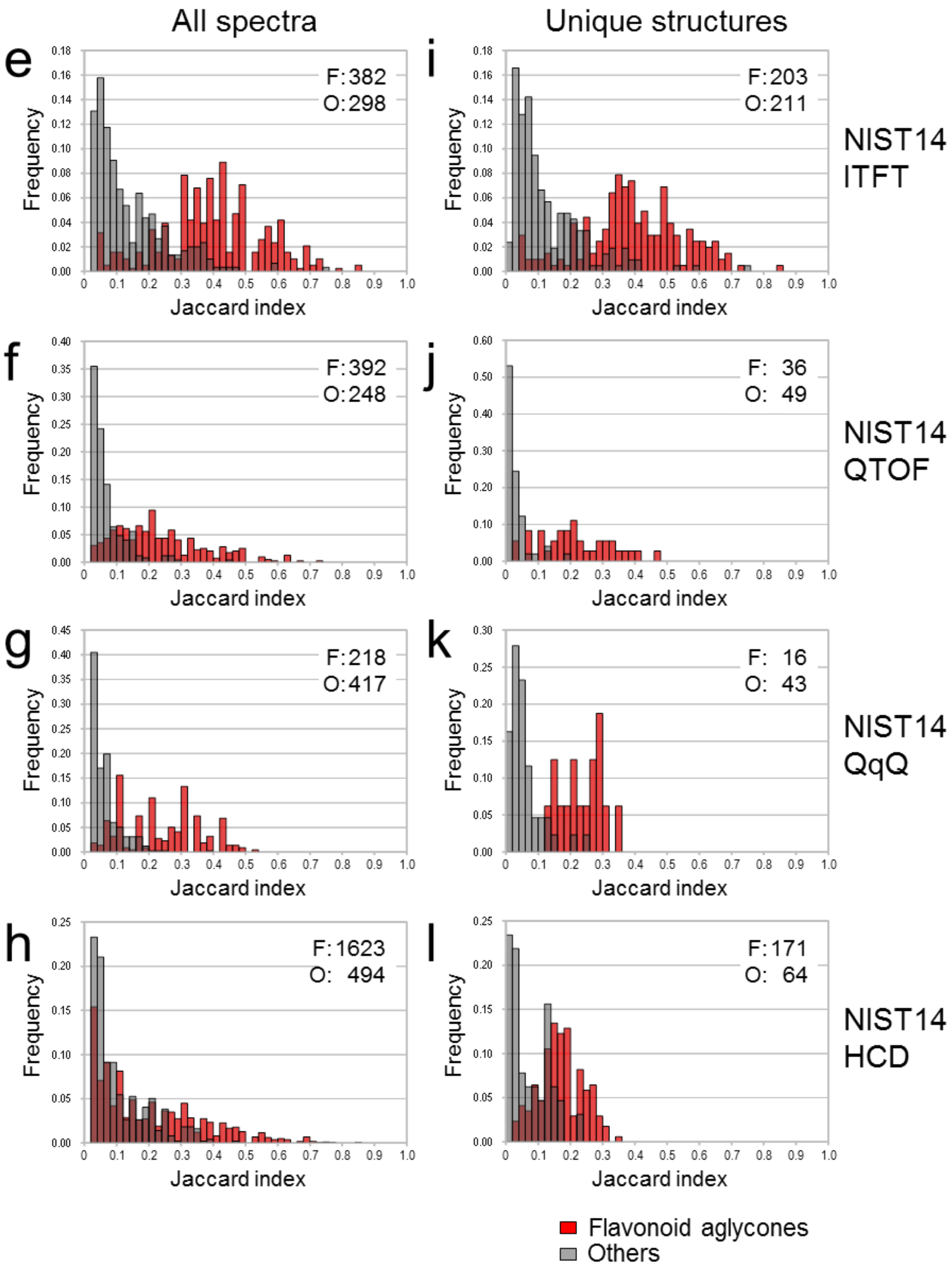

Supplementary Figure S2. Frequency distribution of spectra with various Jaccard indices from FlavonoidSearch. *Continued.*

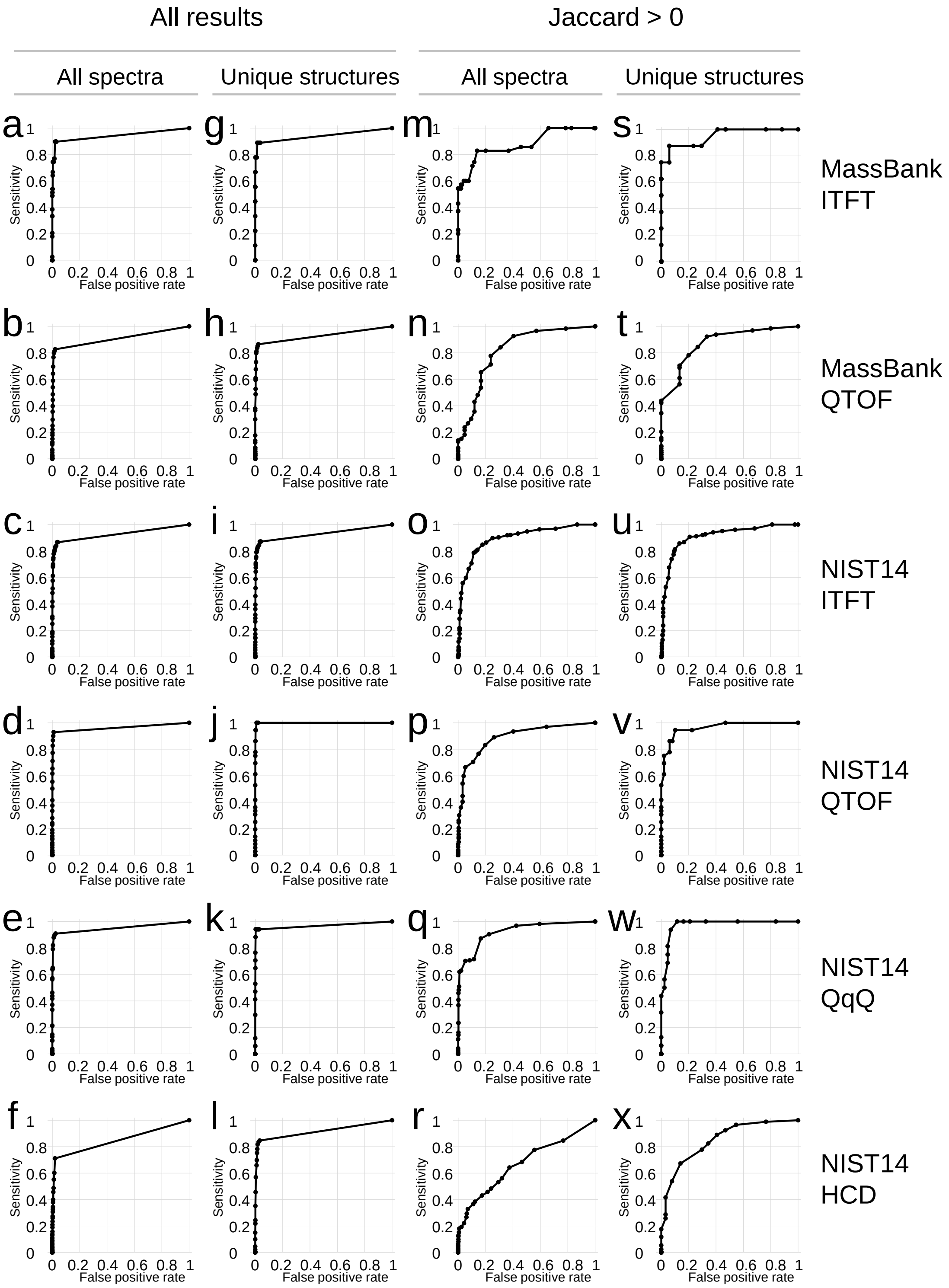

Supplementary Figure S3. Receiver operator characteristic (ROC) curves for the discrimination of flavonoid aglycones. The ROC curves were constructed using all the results (**a**–**l**, 'All results' columns) and only the results that showed Jaccard indices greater than zero (**m**–**x**, 'Jaccard > 0' columns). To remove bias from repeat measurements of the same compound, unique structures were extracted using the first block of InChIKey, and these results are shown in the 'Unique structures' columns (**g**–**l** and **s**–**x**). The average

Jaccard index from the repeat measurements was used for the calculation. The values of the areas under the curves are given in **Supplementary Tables S13** and **S15**. Data were obtained from MassBank (ion trap/Fourier transform MS (ITFT), **a**, **g**, **m** and **s**; and quadrupole time-of-flight MS (QTOF), **b**, **h**, **n** and **t**), and NIST14 (IT/FT (ITFT), **c**, **i**, **o** and **u**; QTOF, **d**, **j**, **p** and **v**; triple quadrupole MS (QqQ), **e**, **k**, **q** and **w**; and high-energy collisional dissociation MS (HCD), **f**, **l**, **r** and **x**).

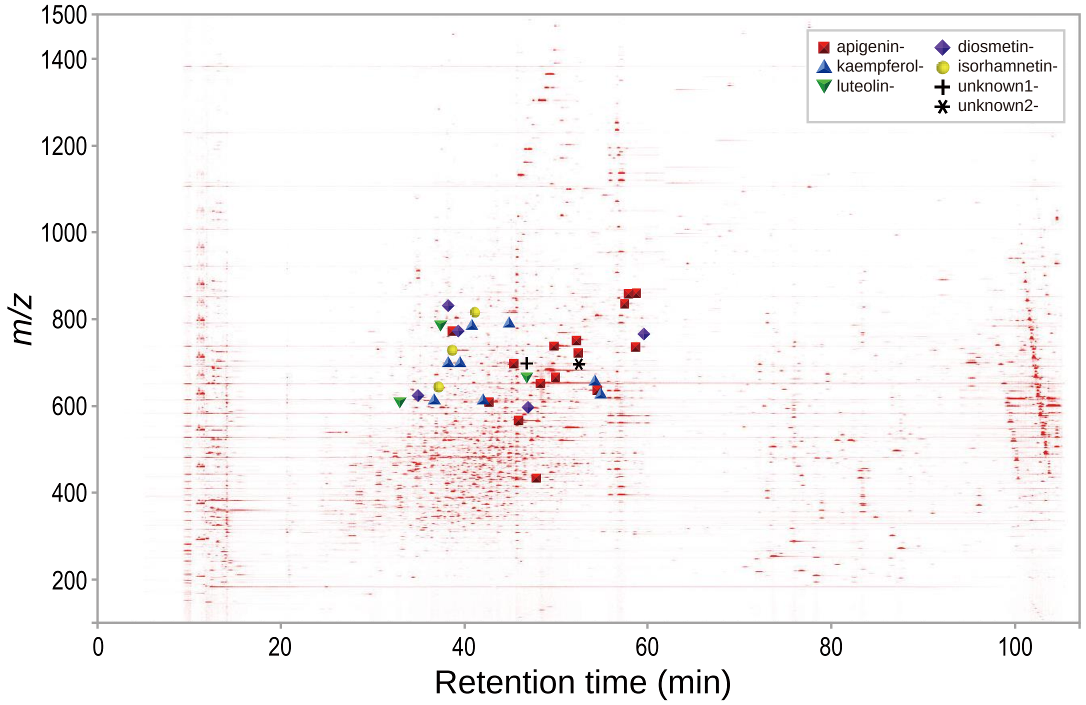

Supplementary Figure S4. Flavonoids in parsley samples (an overall view of Figure 4a). The positions (retention time and *m/z*) of flavonoids in parsley which were annotated by FlavonoidSearch (Jaccard index >0.3) and manual curation were plotted on a two-dimensional mass chromatogram. Peaks of derivatives of characteristic aglycones in parsley (apigenin derivatives, red squares; kaempferol derivatives, blue triangles; luteolin derivatives, green inverted triangles; diosmetin derivatives, purple diamonds and isorhamnetin derivatives, yellow circles) and two unknown aglycones ($C_{16}H_{13}O_7^+$, plus symbol; and $C_{17}H_{15}O_6^+$, asterisk) are represented.

Supplementary Table S4. Trends in the intensities of the fragment ions.

MSMS-category	IHD [a]	Substituents on the A- and/or B-rings																	
		OH		OH, OMe			OH, Prenyl			OH, OMe, Prenyl				OH, Pyran			OH, *C*-Glucosyl		
		C-ring cleavage	Small neutral loss	C-ring cleavage	Small neutral loss	Neutral loss of Methoxyl	C-ring cleavage	Small neutral loss	Neutral losses of Prenyl	C-ring cleavage	Small neutral loss	Neutral loss of Methoxyl	Neutral losses of Prenyl	C-ring cleavage	Small neutral loss	Neutral losses of Pyran	C-ring cleavage	Small neutral loss	Neutral losses of *C*-Glucosyl
Coumestan	11.5	-	++	-	++	+													
Anthocyanidin	10.5	+	+	+	+	++													
Isoflavone		+	+	+	+	++													
Flavonol		+	+	+	+	++													
Flavone		+	+	-	-	++											-	-	++
Aurone		+	+																
4-Phenylcoumarin		+	++																
Chalcone	9.5	++	+	++	+	+													
2'-Hydroxychalcone		++	+	++	+	-													
Flavanone		++	+	++	+	-				+	+	-	++						
Dihydroflavonol		+	++																
Isoflavanone		++	++	++	++	-													
Pterocarpan				++	+	+	++	+	+					++	+	+			
Isoflavan	8.5	++	-	++	-	-													
Flavan-3-ol		++	-																
Dihydrochalcone		++	-	++	-	-													

A summary of the intensities of the fragment ions observed in each MSMS-category for different combinations of substituents on the A- and/or B-rings (OH, OMe, prenyl, pyran and *C*-glucosyl) and sources of ions (derived from C-ring cleavage, small neutral loss, neutral loss of OMe and *C*-type substituents [prenyl, pyran and *C*-glucosyl]). Intensities were also evalauted for the precursor ions but these results are not shown. 'Pyran' is used to represent the [2,3:m,n]-(6,6-dimethylpyrano) group.

'++' indicates that a majority of the fragment ions were detected at high intensity (similar to that of the base peak).

'+' indicates that a majority of the fragment ions were detected at low intensity (less than half that of the base peak).

'–' indicates that no fragment ion was detected.

Blank cells are for standard compounds that were not measured in this work.

[a] The index of hydrogen deficiency calculated for the positively charged backbone structure.

Supplementary Table S10. Parameter settings for the search tools used in the evaluation of accuracy and flavonoid discrimination power.

Search tool	Compound database	Polarity	Adduct type	Limit number of results	Mass tolerance for precursor ion	Mass tolerance for fragment ion [a]					
						In-house measured standard compounds			MassBank	NIST14	
						FT of LTQ-FT	IT of LTQ-FT	IT of LTQ-Orbitrap		ITFT, QqQ	QTOF, HCD
**FsTool (this study)**	FsDatabase (this study)	positive	$[M+H]^+$	-	0.01 Da	0.01 Da	0.2 Da	0.2 Da	0.01 Da	0.2 Da	0.01 Da
**FingerID**	KEGG	positive	-	-	10 ppm	-	-	-	-	-	-
**CFM-ID [b]**	KEGG	positive	$[M+H]^+$	all results	0.01 Da	0.01 Da	0.2 Da	0.2 Da	0.01 Da	0.2 Da	0.01 Da
**MetFrag**	KEGG	positive	$[M+H]^+$	100	0.01 Da	0.01 Da	0.2 Da	0.2 Da	0.01 Da	0.2 Da	0.01 Da
**MetFrag (local SDF) [c]**	FsDatabase (this study) [d]	positive	$[M+H]^+$	100	0.01 Da	0.01 Da	0.2 Da	0.2 Da	0.01 Da	0.2 Da	0.01 Da

'-' represents that the parameter is not required in the tool.

[a] The mass tolerances for fragment ions took into consideration the mass accuracy of the data used as queries.

[b] The same parameters were applied to both scoring functions (Jaccard and DotProduct).

[c] The search mode of MetFrag using user-provided SDF files.

[d] Structural information for MSMS-aglycones with the same mass value as the precursor of the queried data were exported in an SDF file and used as a target database.

Supplementary Table S11. Number of query spectra and mean number of candidate molecules in the search results for each dataset.

	In-house measured standard compounds		MassBank		NIST14			
	LTQ-FT	LTQ-Orbitrap	ITFT	QTOF	ITFT	QTOF	QqQ	HCD
**All compounds**								
				**numbers of queries**				
spectra	89	88	35	208	107	312	78	476
unique structures [a]	84	85	8	47	62	27	6	39
				**mean number of candidates**				
FlavonoidSearch	27.71	26.43	22.06	25.53	25.21	22.63	25.05	26.24
FlavonoidSearch (KEGG) [b]	11.96	11.83	13.31	12.49	13.02	13.52	11.72	12.67
CFM-ID	9.65	10.13	12.26	11.21	11.39	12.79	9.63	10.58
MetFrag	9.78	9.55	11.89	11.51	11.30	12.35	9.21	10.70
MetFrag (local SDF) [c]	24.52	25.41	20.89	24.40	24.07	21.68	24.51	25.22
FingerID	8.57	8.50	11.06	10.21	10.35	11.25	8.27	9.69
**Common compounds** [d]								
				**numbers of queries**				
spectra	56	57	35	164	104	312	78	465
unique structures [a]	52	54	8	32	60	27	6	38
				**mean number of candidates**				
FlavonoidSearch	24.41	25.61	22.06	23.29	25.74	22.63	25.05	26.67
FlavonoidSearch (KEGG) [b]	14.16	14.35	14.02	14.64	14.54	14.52	12.90	14.23
CFM-ID	11.59	11.40	12.26	12.46	11.64	12.79	9.63	10.76
MetFrag	11.30	11.23	11.89	12.47	11.42	12.35	9.21	10.74
MetFrag (local SDF) [c]	23.39	24.53	20.89	22.09	24.59	21.68	24.51	25.65
FingerID	10.34	10.25	11.06	11.55	10.49	11.25	8.27	9.75

[a] The number of unique structures was estimated using the first block of the InChIKey.
[b] Results were adjusted to have isobaric molecules in KEGG.
[c] Molecular structures (as a local SDF file) in the FlavonoidSearch were used as target data for the MetFrag search.
[d] Compounds that all tools gave correct results for were chosen.

Supplementary Table S12. Areas under the cumulative curves in the accuracy estimation.

	In-house measured standard compounds			MassBank			NIST14				
	LTQ-FT		LTQ-Orbitrap	ITFT	QTOF	all	ITFT	QTOF	QqQ	HCD	all
	IT	FT	IT								
**All compounds**											
FlavonoidSearch	84%	84%	75%	71%	68%	69%	76%	80%	81%	71%	75%
FlavonoidSearch (KEGG) [a]	**86%**	**87%**	**80%**	**78%**	**83%**	**82%**	**80%**	**86%**	**95%**	**77%**	**81%**
FlavonoidSearch (KEGG Sim) [b]	50%	50%	47%	52%	48%	49%	49%	54%	73%	50%	53%
CFM-ID (DotProduct)	74%	74%	72%	61%	73%	71%	74%	73%	75%	67%	70%
CFM-ID (Jaccard)	66%	66%	62%	53%	58%	57%	63%	68%	67%	58%	63%
MetFrag	66%	57%	56%	58%	56%	57%	60%	53%	46%	52%	53%
MetFrag (local SDF) [c]	58%	55%	56%	61%	57%	57%	58%	60%	84%	57%	60%
FingerID	55%	54%	51%	42%	55%	53%	51%	49%	45%	45%	47%
**Common compounds** [d]											
FlavonoidSearch	86%	85%	83%	71%	74%	73%	76%	80%	81%	71%	75%
FlavonoidSearch (KEGG) [a]	**88%**	**88%**	**86%**	**86%**	**86%**	**86%**	**84%**	**88%**	**95%**	**81%**	**85%**
FlavonoidSearch (KEGG Sim) [b]	50%	50%	47%	52%	48%	49%	48%	54%	73%	49%	53%
CFM-ID (DotProduct)	74%	74%	73%	61%	73%	71%	74%	73%	75%	67%	70%
CFM-ID (Jaccard)	66%	66%	63%	53%	58%	57%	63%	68%	67%	58%	63%
MetFrag	65%	55%	57%	58%	56%	57%	60%	53%	46%	51%	52%
MetFrag (local SDF) [c]	58%	53%	56%	61%	56%	57%	57%	60%	84%	56%	60%
FingerID	53%	53%	52%	42%	55%	52%	51%	49%	45%	46%	47%

The areas under the cumulative curves for Supplementary Figure S2. The highest value in each dataset is shown in bold.

[a] Results were adjusted to have isobaric molecules in KEGG.

[b] Results were calculated assuming that all flavonoids in candidates of FlavonoidSearch (KEGG) search were correct regardless of their Jaccard index.

[c] Molecular structures (as a local SDF file) in the FlavonoidSearch were used as target data for the MetFrag search.

[d] Compounds that all tools gave correct results for were chosen.

Supplementary Table S13. Statistics for discrimination of flavonoid aglycones using the Jaccard index from FlavonoidSearch.

	MassBank				NIST							
	All spectra		Unique structure [a]		All spectra				Unique structure [a]			
	ITFT	QTOF	ITFT	QTOF	ITFT	QTOF	QqQ	HCD	ITFT	QTOF	QqQ	HCD
**Flavonoid aglycones**	39	282	9	74	441	422	240	2282	233	36	17	202
**Non-flavonoid aglycones**	4912	2058	461	681	7492	23647	17524	25403	5108	2364	1496	1963
**total number**	4951	2340	470	755	7933	24069	17764	27685	5341	2400	1513	2165
**True Positives**	35	233	8	64	382	392	218	1623	203	36	16	171
**False Negatives**	4	49	1	10	59	30	22	659	30	0	1	31
**False Positives**	144	42	17	15	298	248	417	494	211	49	43	64
**True Negatives**	4768	2016	444	666	7194	23399	17107	24909	4897	2315	1453	1899
**Sensitivity**	0.897	0.826	0.889	0.865	0.866	0.929	0.908	0.711	0.871	1.000	0.941	0.847
**Specificity**	0.971	0.980	0.963	0.978	0.960	0.990	0.976	0.981	0.959	0.979	0.971	0.967
**False Positive Rate**	0.103	0.174	0.111	0.135	0.134	0.071	0.092	0.289	0.129	0.000	0.059	0.153
**False Negative Rate**	0.029	0.020	0.037	0.022	0.040	0.010	0.024	0.019	0.041	0.021	0.029	0.033
**AUC**	0.944	0.908	0.941	0.929	0.927	0.963	0.951	0.848	0.930	0.999	0.969	0.917

When there is at least one candidate with a Jaccard index greater than zero in the FlavonoidSearch result, the query is classed as a flavonoid aglycone.

[a] Unique structure number was estimated by identity of the first block of InChIKey. A mean value of Jaccard scores for molecules having the same structure is used for judgement.

Supplementary Table S14. False negatives and their predicted fragments in the FlavonoidSearch database.

Predicted fragments?	MassBank		NIST			
	ITFT	QTOF	ITFT	QTOF	QqQ	HCD
**No**	4<br>(1)	23<br>(9)	55<br>(28)	0<br>(0)	22<br>(3)	273<br>(27)
**Yes**	0<br>(0)	26<br>(18)	4<br>(2)	30<br>(6)	0<br>(0)	386<br>(131)
**total**	4	49	59	30	22	659

For false negatives that arose in the discrimination of flavonoid aglycones using the Jaccard index, this table details the number of spectra with and without predicted fragments for the original compounds in the FlavonoidSearch. False negatives observed in Supplementary Table S13 (all spectra) are shown. Values in parentheses show the numbers of unique structures estimated using the first block of the InChIKey.

Supplementary Table S15. Maximum accuracy and Youden index for the discrimination of flavonoid aglycones using the Jaccard index from FlavonoidSearch.

	**MassBank**				**NIST**							
	**All spectra**		**Unique structure [a]**		**All spectra**				**Unique structure [a]**			
	**ITFT**	**QTOF**	**ITFT**	**QTOF**	**ITFT**	**QTOF**	**QqQ**	**HCD**	**ITFT**	**QTOF**	**QqQ**	**HCD**
Maximum accuracy	0.911	0.884	0.920	0.873	0.837	0.831	0.863	0.767	0.862	0.918	0.932	0.826
	(0.41)	(0.07)	(0.19)	(0.07)	(0.25)	(0.09)	(0.17)	(0.01)	(0.25)	(0.07)	(0.15)	(0.05)
Maximum Youden index	0.690	0.539	0.816	0.589	0.671	0.634	0.706	0.268	0.724	0.842	0.884	0.532
	(0.21)	(0.13)	(0.19)	(0.09)	(0.31)	(0.11)	(0.11)	(0.11)	(0.25)	(0.07)	(0.13)	(0.15)
Flavonoid aglycones	35	233	8	64	382	392	218	1623	203	36	16	171
Non-flavonoid aglycones	144	42	17	15	298	248	417	494	211	49	43	64
Total number	179	275	25	79	680	640	635	2117	414	85	59	235
**Statistics at Max. Youden Index**												
True Positive	29	181	7	59	300	326	190	1042	174	34	16	115
False Negative	6	52	1	5	82	66	28	581	29	2	0	56
False Positive	20	10	1	5	34	49	69	185	28	5	5	9
True Negative	124	32	16	10	264	199	348	309	183	44	38	55
Sensitivity	0.829	0.777	0.875	0.922	0.785	0.832	0.872	0.642	0.857	0.944	1.000	0.673
Specificity	0.861	0.762	0.941	0.667	0.886	0.802	0.835	0.626	0.867	0.898	0.884	0.859
False Negative Rate	0.171	0.223	0.125	0.078	0.215	0.168	0.128	0.358	0.143	0.056	0.000	0.327
False Positive Rate	0.139	0.238	0.059	0.333	0.114	0.198	0.165	0.374	0.133	0.102	0.116	0.141
Area Under the Curve	0.877	0.821	0.949	0.874	0.898	0.893	0.923	0.671	0.913	0.963	0.975	0.848

The maximum accuracy and the maximum Youden index were estimated using the search results with Jaccard indices greater than zero. The values in parentheses indicate the median value of the Jaccard index in the range (± 0.01) where the maximum value is observed.

[a] The number of unique structures was estimated using the first block of the InChIKey. The mean value of Jaccard indices for molecules with the same structure was used for the calculation.

Supplementary Table S18. Combination of *O*-substituents and MSMS-aglycones detected in parsley.

Annotated *O*-Substituent	known/unknown in metabolomics.jp	Annotated MSMS-aglycone						
		Apigenin	Kaempferol	Luteolin	Diosmetin	Isorhamnetin	unknown 1 ($C_{16}H_{13}O_7^+$)	unknown 2 ($C_{17}H_{15}O_6^+$)
Hex	known	1						
Hex+$C_{13}H_{20}O_7$	unknown	1						
Hex+Feruloyl	known		1					
Hex+Glucuronosyl	known	1						
Hex+Hex	known		2	1	1	1		
Hex+Hex+(Butyroyl or Isobutyroyl)	unknown	1						
Hex+Hex+Coumaroyl	known				1			
Hex+Hex+Feruloyl	known			1				
Hex+Hex+Feruloyl or Hex+Pen+Sinapoyl	known	1	1					
Hex+Hex+Malonyl	known		2			1		
Hex+Hex+Malonyl+(Butyroyl or Isobutyroyl)	unknown	1						
Hex+Hex+Malonyl+$C_9H_{12}O_2$	unknown	1						
Hex+Hex+Malonyl+Malonyl	known		1			1		
Hex+Hex+Sinapoyl	known				1			
Hex+Malonyl+$C_{10}H_{16}O_5$	unknown	1			1			
Hex+Malonyl+$C_{17}H_{22}O_7$	unknown	1						
Hex+Malonyl+$C_{17}H_{24}O_7$	unknown	1						
Hex+Malonyl+Glucuronosyl	known	1						
Hex+Pen	known	1			1			
Hex+Pen+(Butyroyl or Isobutyroyl)	unknown	1						
Hex+Pen+Malonyl	known	1		1			1	1
Hex+Pen+Malonyl+Malonyl	unknown	1						
Hex+Sinapoyl	known		1					

The figures in the columns are the number of flavonoid peaks annotated in parsley (Supplementary Table S17).

References

1 Marston, A. & Hostettmann, K. in *Flavonoids: Chemistry, Biochemistry and Applications*. (eds Ø.M. Andersen & K.R. Markham) Ch. 1, 1-36 (CRC Press Tayler & Francis Group, 2006).

2 de Rijke, E. *et al.* Analytical separation and detection methods for flavonoids. *J. Chromatogr. A* **1112**, 31-63 (2006).

3 Ma, Y. L., Li, Q. M., Van den Heuvel, H. & Claeys, M. Characterization of flavone and flavonol aglycones by collision-induced dissociation tandem mass spectrometry. *Rapid Commun. Mass Spectrom.* **11**, 1357-1364 (1997).

4 Stevens, J. F. *et al.* Leaf surface flavonoids of Chrysothamnus. *Phytochemistry* **51**, 771-780 (1999).

5 Justino, G. C., Borges, C. M. & Florêncio, M. H. Electrospray ionization tandem mass spectrometry fragmentation of protonated flavone and flavonol aglycones: a re-examination. *Rapid Commun. Mass Spectrom.* **23**, 237-248 (2009).

6 Kuhn, F., Oehme, M., Romero, F., Abou-Mansour, E. & Tabacchi, R. Differentiation of isomeric flavone/isoflavone aglycones by MS^2 ion trap mass spectrometry and a double neutral loss of CO. *Rapid Commun. Mass Spectrom.* **17**, 1941-1949 (2003).

7 Antignac, J.-P., Cariou, R., Le Bizec, B., Cravedi, J.-P. & Andre, F. Identification of phytoestrogens in bovine milk using liquid chromatography/electrospray tandem mass spectrometry. *Rapid Commun. Mass Spectrom.* **17**, 1256-1264 (2003).

8 Oliveira, M. C., Esperança, P. & Almoster Ferreira, M. A. Characterisation of anthocyanidins by electrospray ionisation and collision - induced dissociation tandem mass spectrometry. *Rapid Commun. Mass Spectrom.* **15**, 1525-1532 (2001).

9 Zhang, L. *et al.* Characterization of flavonoids in the extract of Sophora flavescens Ait. by high-performance liquid chromatography coupled with diode-array detector and electrospray ionization mass spectrometry. *J. Pharm. Biomed. Anal.* **44**, 1019-1028 (2007).

10 Nakayama, M. *et al.* Mass spectra of pterocarpan derivatives. *J. Mass Spectrom. Soc. Jpn.* **20**, 239-247 (1972).

11 Simons, R., Vincken, J. P., Bakx, E. J., Verbruggen, M. A. & Gruppen, H. A rapid screening method for prenylated flavonoids with ultra-high-performance liquid chromatography/electrospray ionisation mass spectrometry in licorice root extracts. *Rapid Commun. Mass Spectrom.* **23**, 3083-3093 (2009).

12 Tóth, E., Dinya, Z. & Antus, S. Mass spectrometric studies of the pterocarpan skeleton. *Rapid Commun. Mass Spectrom.* **14**, 2367-2372 (2000).

13 Tai, Y., Pei, S., Wan, J., Cao, X. & Pan, Y. Fragmentation study of protonated

chalcones by atmospheric pressure chemical ionization and tandem mass spectrometry. *Rapid Commun. Mass Spectrom.* **20**, 994-1000 (2006).

14 Zhang, J. & Brodbelt, J. S. Structural characterization and isomer differentiation of chalcones by electrospray ionization tandem mass spectrometry. *J. Mass Spectrom.* **38**, 555-572 (2003).

15 Ardanaz, C. E., Traldi, P., Vettori, U., Kavka, J. & Guidugli, F. The ion-trap mass spectrometer in ion structure studies. The case of $[M-H]^+$ ions from chalcone. *Rapid Commun. Mass Spectrom.* **5**, 5-10 (1991).

16 Zeeb, D. J., Nelson, B. C., Albert, K. & Dalluge, J. J. Separation and identification of twelve catechins in tea using liquid chromatography/atmospheric pressure chemical ionization-mass spectrometry. *Anal. Chem.* **72**, 5020-5026 (2000).

17 Cuyckens, F. & Claeys, M. Mass spectrometry in the structural analysis of flavonoids. *J. Mass Spectrom.* **39**, 1-15 (2004).

18 Cavaliere, C., Foglia, P., Pastorini, E., Samperi, R. & Laganà, A. Identification and mass spectrometric characterization of glycosylated flavonoids in Triticum durum plants by high-performance liquid chromatography with tandem mass spectrometry. *Rapid Commun. Mass Spectrom.* **19**, 3143-3158 (2005).

19 Stobiecki, M. Application of mass spectrometry for identification and structural studies of flavonoid glycosides. *Phytochemistry* **54**, 237-256 (2000).

20 Balza, F., Crins, W. J., Bohm, B. A. & Towers, G. H. N. Mass spectrometry in the differentiation of flavanones and dihydroflavonols. *Phytochemistry* **27**, 2715-2717 (1988).

21 Wolfender, J. L. *et al.* Evaluation of Q-TOF-MS/MS and multiple stage IT-MS^n for the dereplication of flavonoids and related compounds in crude plant extracts. *Analusis* **28**, 895-906 (2000).

22 Horai, H. *et al.* MassBank: a public repository for sharing mass spectral data for life sciences. *J. Mass Spectrom.* **45**, 703-714 (2010).

23 Allen, F., Pon, A., Wilson, M., Greiner, R. & Wishart, D. CFM-ID: a web server for annotation, spectrum prediction and metabolite identification from tandem mass spectra. *Nucleic Acids Res.* **42**, W94-99 (2014).

24 Heinonen, M., Shen, H., Zamboni, N. & Rousu, J. Metabolite identification and molecular fingerprint prediction through machine learning. *Bioinformatics* **28**, 2333-2341 (2012).

25 Wolf, S., Schmidt, S., Müller-Hannemann, M. & Neumann, S. In silico fragmentation for computer assisted identification of metabolite mass spectra. *BMC Bioinformatics* **11**, 148 (2010).

26 de Laeter, J. R. *et al.* Atomic weights of the elements: review 2000 (IUPAC technical report). *Pure Appl. Chem.* **75**, 683-800 (2003).
27 Sakurai, N. *et al.* Tools and databases of the KOMICS web portal for preprocessing, mining, and dissemination of metabolomics data. *BioMed Res. Int.* **2014**, 1-11 (2014).
28 Ara, T. *et al.* Metabolonote: a wiki-based database for managing hierarchical metadata of metabolome analyses. *Front. Bioeng. Biotechnol.* **3** (2015).
